# Structure and Diversity of the Rhesus Macaque Immunoglobulin Loci through Multiple *De Novo* Genome Assemblies

**DOI:** 10.3389/fimmu.2017.01407

**Published:** 2017-10-27

**Authors:** Akshaya Ramesh, Sam Darko, Axin Hua, Glenn Overman, Amy Ransier, Joseph R. Francica, Ashley Trama, Georgia D. Tomaras, Barton F. Haynes, Daniel C. Douek, Thomas B. Kepler

**Affiliations:** ^1^Graduate Program in Genetics and Genomics, Boston University School of Medicine, Boston, MA, United States; ^2^NIH Vaccine Research Center, National Institute of Allergy and Infectious Diseases, National Institutes of Health, Bethesda, MD, United States; ^3^Department of Microbiology, Boston University School of Medicine, Boston, MA, United States; ^4^Duke Human Vaccine Institute, Duke University Medical Center, Durham, NC, United States; ^5^Department of Mathematics and Statistics, Boston University, Boston, MA, United States

**Keywords:** rhesus macaque, immunoglobulin, allelic diversity, high-throughput sequencing, evolution, vaccine

## Abstract

The rhesus macaque is a critically important animal model in biomedical research, most recently playing a key role in the development of vaccines against human immunodeficiency virus-1. Nevertheless, the immunoglobulin (Ig) loci of macaques are as yet incompletely determined and our understanding of differences between human and macaque humoral immunity remains deficient. We completed a high-coverage, high-quality whole genome sequencing and assembly project with a single rhesus macaque of Indian origin, and partial genome assemblies using genomic molecular targeting of the Ig loci in nine other rhesus macaques of Indian origin. These data indicate that the macaque Ig loci are substantially more diverse than those in humans, including greater sequence diversity and copy-number variation between individuals. It appears likely that such copy-number variation even occurs between allelic loci within individuals. Different Ig gene families in the macaque show distinct relationships to the corresponding human gene families and appear to evolve under different mechanisms. These results raise intriguing questions about the evolution of antigen receptors in primates but also have important practical implications for the design and interpretation of biomedical studies.

## Introduction

The rhesus macaque (*Macaca mulatta*) is one of the most important animal models in biomedicine and has been instrumental in the study of human infectious diseases for decades (1, 2). It has played a key role in the development of vaccines against several infectious agents including *Influenzavirus, Variola, Yersinia pestis*, and *Plasmodium* spp (2). Recently, they have become the focus of increased attention as a critical component in the quest for a vaccine against human immunodeficiency virus-1 (HIV-1). Macaques infected with simian immunodeficiency virus or the artificial hybrid virus (SHIV) are the best animal models currently available for HIV/AIDS studies and vaccine research. Considering their crucial role in vaccinology, it is imperative that the genetics of the macaque immune system is thoroughly understood.

Most vaccines provide protection through the sensing and effector functions of antibodies. The efficacy of the vaccine antibody response depends on, among other things, the availability of antibodies of the appropriate binding specificity and their development into high-affinity antigen-binding molecules through affinity maturation. The availability of specific antibodies is attributable to the formation of immunoglobulin variable-region genes (IgVRG) by the stochastic recombination of germline gene segments arrayed within each of the three immunoglobulin (Ig) loci: IGH, IGK, and IGL. Each IgVRG is assembled from two (IGK, IGL) or three (IGH) gene segments selected stochastically, with junctional diversity in the form of variable recombination points and the addition of non-templated nucleotides. These gene segments are denoted *Variable* (V), *Joining* (J), and, in the IGH locus only, *Diversity* (D). Post-exposure antibody specificity is refined through affinity maturation, which acts through the random somatic mutation of IgVRG and natural selection favoring those B cells that thus acquire an increased affinity for the eliciting antigen. Affinity maturation is essential for the generation of protective humoral responses.

Allelic diversity among the gene segments, including copy-number polymorphism, further enhances the diversity of the Ig repertoire at the host population level (3, 4). Given the prominent role of affinity maturation for effective vaccine responses, it is important to distinguish IgVRG somatic mutations from allelic polymorphisms in the analysis of the development of the post-vaccination B cell response.

Recently, high throughput sequencing approaches have allowed sequencing of the complete IgVRG repertoire, providing large amounts of data—and potentially enormously useful information—on vaccine-induced affinity maturation. Over the last decade, researchers have isolated potent broadly neutralizing antibodies (BNAbs) against HIV-1 that neutralize a broad spectrum of virus variants (5–15). These antibodies typically exhibit extremely high levels of somatic mutation and strong biases in the utilization of Variable (V), Diversity (D), and Joining (J) genes. In order for these insights to be realized in vaccine studies in macaques, somatic mutations must be identified reliably, and the correspondence among gene segments in humans and macaques established. The research reported here was motived largely by the urgent need to address these problems.

Though the first whole genome sequence (WGS) for the rhesus macaque was published in 2007 (16), our understanding of the Ig loci remains limited. A detailed characterization of the Ig region in macaques is crucial to the understanding the applicability to humans of studies of adaptive immunity in macaques. Macaque genomic sequence data are incomplete, and the Ig locus itself is complex and highly repetitive. In addition, the existing WGS of the macaque is poor quality, with over 50% of the genome misannotated due to several contig misassemblies and sequencing errors (17). To address this issue, we have assembled the Ig loci at high-coverage from the genome of one Indian-origin rhesus macaque designated M0. These loci will be referred to as the *reference* loci. To examine allelic diversity, we also sequenced and assembled the Ig loci from the genomes of nine additional Indian rhesus macaques designated M1–M9. We believe that understanding the immunogenetics of this important non-human primate is vital to the development of effective vaccines and to optimizing the use of macaques as viable models for adaptive immunity.

## Materials and Methods

### WGS Library Preparation

In order to obtain the complete Ig locus, we sequenced the whole genome of the macaque using two complementary, Illumina sequencing strategies. Genomic DNA was extracted from macaque ovum of an Indian-origin rhesus macaque (Central State Primate colony) using the Qiagen Blood and Tissue kit to obtain a concentration of 101 ng/µl of DNA. Libraries of three insert sizes (standard: 400 bp, mate pairs: 3,000–5,000 bp, 8,000–11,000 bp) were constructed and sequenced in a paired end manner on an Illumina HiSeq2500 at 110X coverage. We will refer to reads obtained from this strategy as PMPs (Paired end and Mate Pair reads). Second, we used the Illumina TruSeq technology to obtain long reads (1,500–20,000 bp) for the WGS of the Rhesus Macaque. Genomic DNA was extracted from the same Indian-origin macaque and the libraries were prepared and sequenced using the Illumina TruSeq technology. We will refer to reads obtained from this strategy as SLRs (Synthetic Long Reads).

### Assembly of Reference Ig Loci from Unbiased Whole-Genome Sequencing

We used multiple genome assembly strategies to assemble the reference locus and in the following section, we describe the genome assembly strategy that produced the most reliable result for each locus.

### IGH Assembly

To ensure that the reads obtained from the Illumina runs (PMPs and SLRs) were of good quality, we performed quality control of the reads using the FASTQC v0.10.1 (18) toolkit. All low quality reads (bases with PHRED < 20) and adapter sequences were clipped using Trimmomatic v0.32 (19) and the unbiased, trimmed reads were used as inputs for genome assembly. We used SOAP*denovo*2 (20), a de-brujin graph assembler, to assemble all the PMPs representing the whole genome of the rhesus macaque. We optimized the parameters for the assembly, and the optimal assembly (K-mer = 69) was chosen using assembly statistics generated by QUASTv2.3 (21). The gaps in the assembly were closed using GapCloser v1.12 (20) and contigs less than 1,000 bp were discarded. Next, we scaffolded the assembly using PMPS—SSPACE-standardv3.0 (22) and SLRs—SSPACE-LongReadv1.1 (23). Finally, we used transcriptome data (16) and scaffolded the assembly using L_RNA_scaffolder (24). We used African green monkey, cynomolgus macaque and human IGH as queries in BLAST+ to obtain the contigs representing the rhesus macaque IGH locus.

### IGL and IGK Assembly

We used Celerav8.1 to assemble all the SLRs generated from Illumina TruSeq. Reads obtained using the TruSeq strategy have been shown to have low error rates. As the SLRs share some characteristics with consensus-corrected PacBio reads, we applied the Celera Assembler parameters recommended for PacBio data to take advantage of the read length and low error rate. We optimized the parameters for the assembly, and the optimal assembly was chosen using assembly statistics generated by QUASTv2.3 (21). The parameters that were used to assemble the genome can be found in Table S9 in Supplementary Material. We used African green monkey, cynomolgus macaque and human IGL and IGK as queries in BLAST+ to obtain the contigs representing the rhesus macaque IGL and IGK locus.

Simultaneously, using African green monkey, Cynomolgus macaque and human IGL and IGK as queries in BLAST+, we identified all the IGL and IGK reads from PMPs dataset. These “baited-Ig” 150 bp reads, along with the IGL and IGK contigs obtained from the SLR assembly were used as inputs for genome assembly using SPAdesv3.0 (25). The short reads were assembled using multiple k-mers 55, 65, 75, 85, 95, 105, and 125, and the optimal assemblies were identified using assembly statistics generated by QUASTv2.3. The optimal assemblies were then combined using Metassembler (26), to obtain a single superior assembly.

### Contig Ordering and Annotation of the Reference Loci

We ordered the assembled contigs against the complete Ig loci of the African green monkey using MUMmerv3.23 (27). The order was assigned based on highest percent identity, contig coverage, and consistent alignment with respect to the query IGH locus—when one of these criteria were inconsistent, the contig order remained undecided. The genes on the Ig locus were identified and annotated based on homology to known Ig genes sequences in non-human primates and humans available through IMGT. The Variable (V) genes in the Ig locus were identified based on identification and evaluation of recombination signals, in-frame coding sequence with invariant Cysteines, leader regions, and splice sites. A gene was classified as functional (F) if it contained all the essential features described above. If the putative V gene had no stop codons in the coding region and intact invariant Cysteines, it was classified as an open reading frame (ORF); if it lacked the other essential features of a V gene and/or has stop codons, we classified it as a non-functional (NF) gene. The Diversity (D) and Joining (J) genes were identified based on identification and evaluation of recombination signals and in-frame coding sequence with the presence of invariant Tryptophan for J genes. If the putative D/J gene had no stop codons in the coding region with intact recombination signals, it was classified as F; other D/J genes lacking these features were classified as NF. The Constant region (C) genes were identified and evaluation of on in-frame coding sequence and splice sites. If the putative C gene had intact in-frame sequence and splice junctions, it was classified as F. C genes lacking these features were classified as NF. The Ig locus maps were generated using MATLABv8.4.0.

### Targeted Sequencing Strategy of Ig Genomic Loci

To obtain further information on the allelic diversity of the Ig locus in the rhesus macaque, we performed targeted Ig sequencing from nine additional macaques. This study was carried out in accordance with National Institute of Health guidelines; all animal procedures were approved by the Vaccine Research Center’s Institutional Animal Care and Use Committee. Agilent molecular “bait” sequences were created by aligning all human Ig and TCR germline sequences from IMGT (28) against the rhesus macaque genome, “rheMac2,” using BLAST+. The contiguous alignment coordinates were then used to extract the appropriate sequences from the genome. These sequences, which represent the Ig and TCR locus on the macaque genome, were then submitted to Agilent to create a custom bait library. The resulting library covered approximately 2MB of the macaque genome with 2× tiled baiting.

Genomic DNA was extracted from skin punches of 9 Indian-origin rhesus macaques (New Caledonia breeding colony) using the Agilent SureSelect gDNA Extraction Kit. 1–3 µg of the gDNA was then sheared using the Covaris E-Series Sample Preparation system into 150–200 bp fragments. gDNA libraries were prepared according to the protocol provided in the SureSelect XT Target Enrichment System Kit for Illumina Multiplexed Sequencing manual. The prepared DNA libraries were then mixed with the SureSelect Capture Library, which consists of the biotinylated RNA library baits that are complimentary to the target region on the prepped library. Streptavidin-coated magnetic beads attach to the baits that have hybridized to the target region and are then magnetically separated from the unbound DNA fraction. The baits were then digested, leaving only single-stranded target DNA. A final PCR was performed to amplify the captured DNA content and to add indices to the target DNA. The samples were then pooled in preparation for 100 bp, paired-end multiplexed sequencing on the Illumina HiSeq platform. Following sequencing, the sequencing reads were trimmed to remove adaptor sequence and low quality bases.

### *De Novo* Assembly and Annotation of the Ig locus

To ensure that the reads obtained from the HiSeq run were of good quality, we carried out quality control of the reads using the FASTQC (18) toolkit. We used the cutadapt toolkit to eliminate poor quality reads (PHRED < 20) and remove adapter sequences. Next, we assembled the reads *de novo* using SPAdes2.4 (25). We optimized the assembly at various k-mer values and selected the optimal assembly using assembly statistics generated by QUAST2.3 (21). Annotation of the Ig locus was carried out in the same manner as described in the previous section.

All annotated sequences are available at Genbank under accession numbers MF989451-MF989952.

### Comparison to the Reference Ig Locus

All Ig genes from the reference loci were used as BLAST + queries against the genes in the baited assemblies. A gene from the baited assemblies was considered an allele of the query if their nucleotide identity was 90% or more, and otherwise treated as a gene not identified in the reference loci.

### Analysis of Diversity

Multiple sequence alignments were generated using ClustalW or MUSCLE in Bioeditv7.0.0 and MEGAv6.0 (29), respectively. Dotplots were generated using YASS (30) with default parameters [scoring matrix for match, transversion, and transition: (5, −4, −3); gap cost (opening): −16; gap cost (extension) −4, window size: 10]. We studied both intraspecies and interspecies diversity of macaques with respect to humans using maximum-likelihood phylogenetics. Maximum likelihood trees were generated using MEGA6.0 (29). The branches on the trees were colored using FigTree and Rainbow Tree. Human Ig genes and alleles that were used in the analysis were downloaded from IMGT. Gene conversion signatures in macaque Ig V genes were identified by manually scanning IGHV, IGLV, and IGKV multiple sequence alignments.

## Results

### Generation of the Reference Ig Loci from Unbiased Whole Genome Sequencing

We used a combination of short reads and long reads generated using next generation sequencing strategies from a rhesus macaque of Indian origin to obtain high-resolution *reference* loci. In addition, we performed baited Ig-sequencing from nine additional macaques of Indian origin and integrated the information from these animals to characterize allelic diversity.

### *IGH* Assembly and Ordering

We obtained 1.8 billion reads (400 bp, standard inserts), 109,184,438 reads (3–5 kb inserts) and 101,348,328 reads (8–11 kb inserts) using Illumina 150 bp, paired-end sequencing representing the whole genome. These reads were assembled using SOAP*denovo*2 (20) (K-mer = 69) (20) and the gaps in the assembly were closed using GapCloserv1.12 (20). Contigs less than 1,000 bp were discarded and the remaining contigs were further scaffolded using SSPACE-standardv3.0 (22). Using the TruSeq strategy, we obtained 9,784,340 SLRs varying in length between 1,500 and 20,000 bp, and these were used to further scaffold the contigs using SSPACE-LongReadv1.1 (23). Finally, we used transcriptome data (16) and scaffolded the assembly using L_RNA_scaffolder (24). 29 scaffolds representing the IGH locus were identified using African green monkey, cynomolgus macaque and human IGH as queries in BLAST+ to obtain the contigs representing the rhesus macaque IGH locus.

The 29 assembled macaque IGH contigs were ordered against the complete IGH locus of the African green monkey using MUMmerv3.23 (27). Based on the alignments of the query to the reference IGH locus, 9 scaffolds were ordered to represent the rhesus macaque IGH locus and 16 scaffolds were additionally ordered to represent a sister IGH locus. Four scaffolds could not be placed. One of the sister IGH contigs, scaffold 5775, had two conflicting coordinates on the African green monkey IGH locus: positions 1,401,047 to 1,454,449 bp or 1,472,966 to 1,575,244 bp. In both these cases, final contig order was unaffected—“sister IGH” of scaffold 5426 (IGH order# 7) or “sister IGH” of scaffold 5426 and 7076 (IGH order #7, #8).

### *IGL* and *IGK* Assembly and Ordering

We assembled the SLRs *de novo* using Celerav8.1 into 130,130 contigs with 26,559 contigs representing 50% of the WGS. Using the African green monkey, cynomolgus macaque and human IGL and IGK as queries in BLAST+, we obtained 2,705 contigs representing the rhesus macaque IGL and IGK locus. Simultaneously, African green monkey, cynomolgus macaque, and human IGL and IGK were also used as queries in bowtie2 (31) against the PMP dataset, to obtain 50,703,106 “baited-Ig” reads. The baited-Ig reads and contigs were used as inputs for genome assembly using SPAdesv3.0 (25), and the optimal assemblies were further combined using Metassembler (26), to obtain 3 and 8 contigs representing the rhesus macaque IGL and IGK locus, respectively.

The eight assembled macaque IGK contigs were ordered against the African green monkey IGK locus to represent 4 IGK and 4 “sister” scaffolds. Similarly, the three assembled macaque IGL contigs were ordered against the African green monkey IGL locus. Data files S1–S3 in Supplementary Material contain the list of all the rhesus IGH, IGK, and IGL contigs aligned against African Green monkey IGH, IGK, and IGL using MUMmerv3.23 and the highlighted region indicates the coordinates that were taken into consideration to determine final IG order.

For all IgVRG+ scaffolds in the reference assembly of M0, IgVRG were located and annotated for the features essential to antibody expression and function (see Materials and Methods). On the basis of these annotations, genes were classified as functional, NF, or ORFs.

### *IGH* 

29 scaffolds were positive for IGH genes. The largest of these is 1.4 Mb long and contains 55 IGHV genes and all 39 IGHD, all 9 IGHJ, and all 9 IGHC genes. A total of 178 IGHV genes (71 functional) belonging to 7 IGHV families were identified across all scaffolds.

### *IGK* 

Eight scaffolds were positive for IGK genes. The largest of these is 744 kb long and contains 61 IGKV genes. A total of 119 IGKV (65 functional), 5 IGKJ genes and 1 IGKC gene were identified.

### *IGL* 

Three scaffolds were positive for IGL genes. The largest of these is 800 kb long and contains 54 IGLV genes, and all seven pairs of alternating IGLJ and IGLC genes. A total of 105 IGLV (46 functional, 1 ORF, 58 NF) were identified across scaffolds.

### Scaffold Ordering

**Figure 1:**
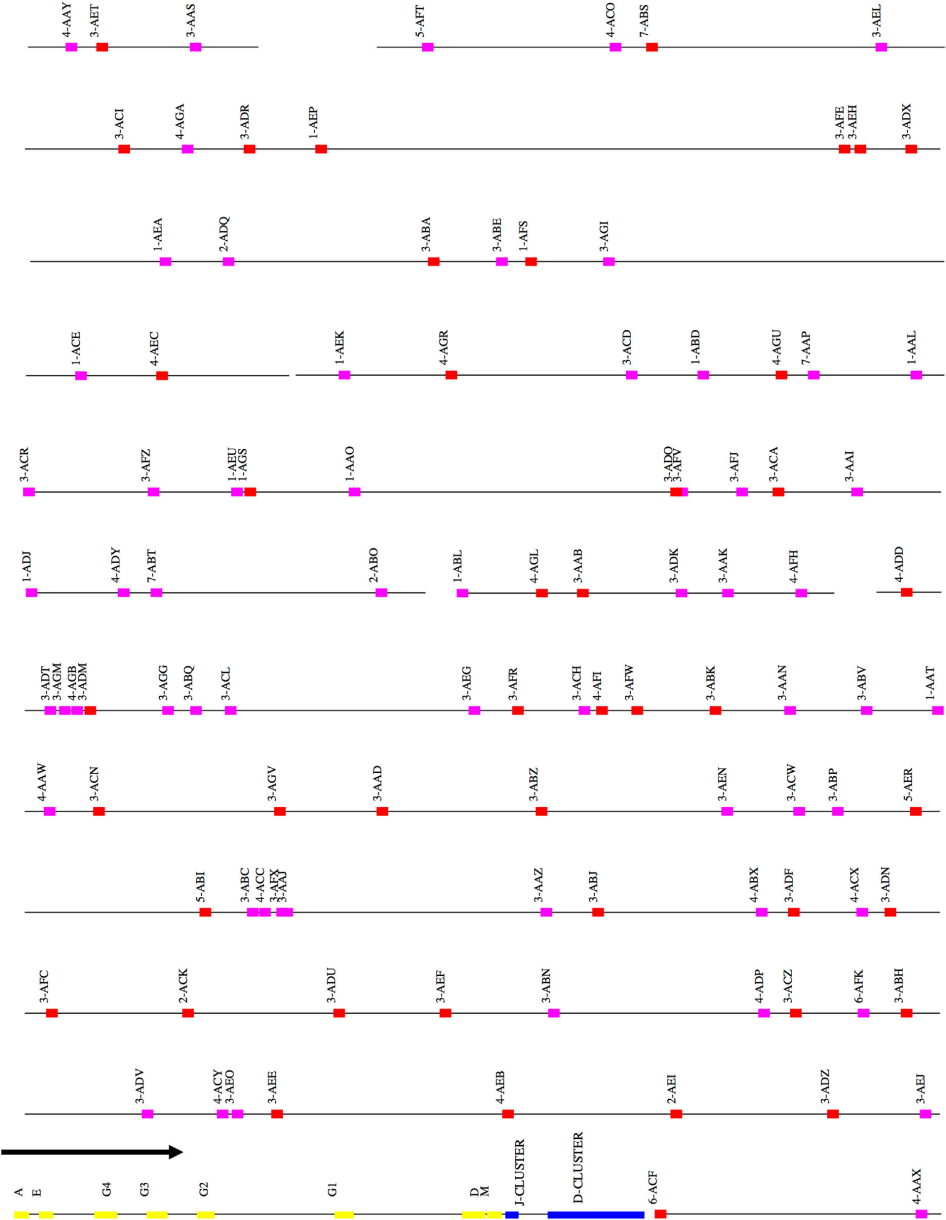
Schematic representation of the rhesus macaque IGH locus. A schematic representation of the rhesus IGH locus (plus strand). The order of the scaffolds was determined by ordering the rhesus macaque IGH scaffolds against the African Green Monkey IGH locus. The functional and non-functional genes are represented in red and magenta blocks, respectively. The J and D clusters are represented in blue blocks and constant region genes in yellow blocks. The constant region scaffold is one sixth the size of the other scaffolds in the figure—this was done to accommodate its large size compared to the IGHV region.

**Figure 2:**
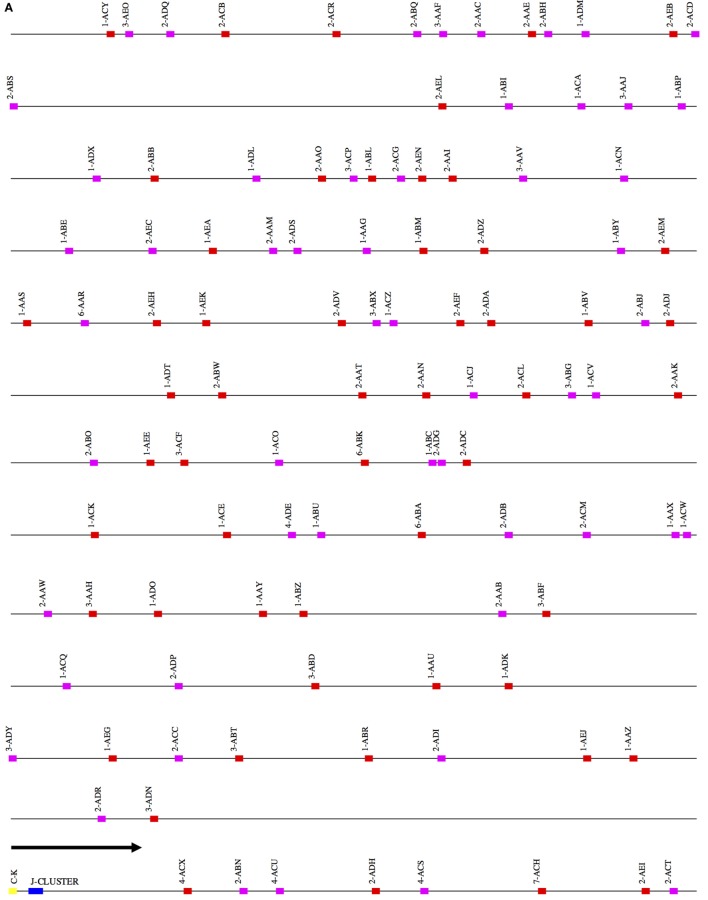

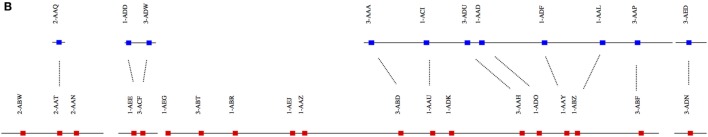
**(A)** Schematic representation of the rhesus macaque IGK locus. A schematic representation of the rhesus IGK locus (plus strand). The order of the scaffolds was determined by ordering the rhesus macaque IGK scaffolds against the African Green Monkey IGK locus. The functional and non-functional genes are represented in red and magenta blocks, respectively. The J cluster and C–K gene are represented in blue and yellow blocks, respectively. **(B)** Copy number variants observed between the main (red) and “sister” (blue) IGK scaffolds in the rhesus macaque. The dots in the figure indicate the correspondence of the “sister” genes and the main scaffolds in IGKV.

**Figure 3:**
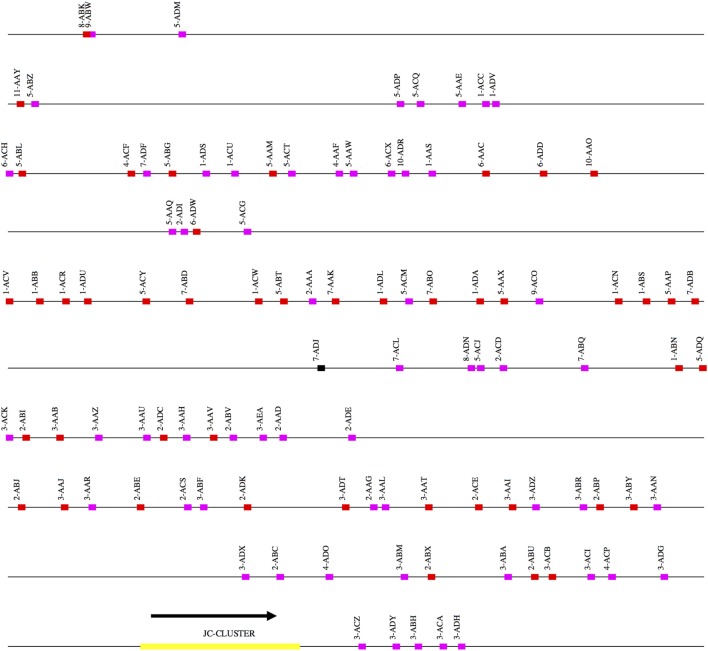
Schematic representation of the rhesus macaque IGL locus. A schematic representation of the rhesus IGK locus (plus strand). The order of the scaffolds was determined by ordering the rhesus macaque IGK scaffolds against the African Green Monkey IGK locus. The functional, non-functional, and open reading frame genes are represented in red, magenta, and black blocks, respectively. The JC cluster is represented as a yellow block.

The scaffolds for each locus were ordered by aligning them against the corresponding loci in the genome assemblies for the African green monkey, *Chlorocebus sabaeus*. The scaffolds thus ordered had substantial overlap. That is, different scaffolds mapped equally well to the same region of the *C. sabaeus* locus. The best explanation for this result is that there is enough polymorphism between the loci on homologous chromosomes to achieve partial resolution. (Further discussion of this point is in the Section “Discussion.”) We have designated the larger of the two ordered scaffold clusters the “main” locus, and the smaller, the “sister” locus. Figures 1–3 represent a schematic of the rhesus Ig locus. Figure 2B represents the “main” and “sister” locus for IGKV.

### *IGHV* 

After ordering the scaffolds, we found that 44 functional IGHV genes localized to the main locus, 20 functional genes to the sister locus, and 7 functional genes to scaffolds that could not be placed (Figure S1A and Table S1A in Supplementary Material).

### IGHD, J, and C

All the IGHD, IGHJ, and IGHC genes were assembled onto one scaffold, along with 55 functional IGHV genes. Thirty-nine functional IGHD genes belonging to six families were identified—of these, ten formed five pairs of identical genes, and three formed one triplet of identical genes (Table S1B in Supplementary Material). These genes are organized into seven homologous clusters, a cluster prototypically containing one gene from each of the IGHD1-6 families. The observed clusters differ from the prototype by deletions and duplications. Nine IGHJ genes (six functional) were identified, with IGHJ5 duplicated. Eight IGHC genes were identified including one IGHA gene and four IGHG genes. An additional pseudogene (IGHEP) was found on an unplaced scaffold.

### *IGKV* 

A total of 119 IGKV genes (Table S2 in Supplementary Material) belonging to 6 IGKV families were identified on four scaffolds constituting the main IGK locus and four contigs constituting the sister locus. We identified 54 functional IGKV genes belonging to six families (Figure S1B in Supplementary Material) and 11 functional genes on the sister IGK locus (Table S2 in Supplementary Material). Functional IGKV genes were identified in both the forward and reverse orientations on the main IGKV scaffolds, with six alternating blocks of homogeneous orientation; the majority—26 and 60 genes—in the two largest such blocks of forward and reverse orientations, respectively.

### IGKJ, *IGKC*

The IGKJ cluster containing four functional genes and one NF gene, and the single IGKC were assembled onto one scaffold, along with 10 IGKV genes.

### *IGLV* 

105 IGLV genes (Table S3 in Supplementary Material) belonging to 11 IGLV families were identified including 46 functional IGLV genes belonging to 10 families (Table S3 and Figure S1C in Supplementary Material) and one ORF belonging to the IGLV7 family. No members of the IGLV9 family were functional.

### IGL-JC

Along with 54 IGLV genes, the IGL-JC cluster was assembled onto one scaffold. Seven pairs of tandem IGLV and IGLC genes were identified. Five of the JC genes were functional. Two of the IGLC genes were identical but were paired with different *IGLJ*.

### Allelic Diversity

#### Generation of Supplementary Assemblies

We performed genomic molecular targeting and sequencing as described (see Materials and Methods) on nine additional macaques (M1–M9) yielding a total of 14.84 (±1.19) million reads with PHRED scores above 28 across their entire length (Table S4 in Supplementary Material). Ig genes were located and annotated as for the reference loci. Comparison to the reference genes was performed by using each reference gene as a query in BLAST+ against the genes in the supplementary assemblies. Because full position information was not typically available in the supplementary assemblies, allelism with respect to the reference assembly was assessed largely by a heuristic based on sequence similarity.

#### *IGHV* 

**Figure 4:**
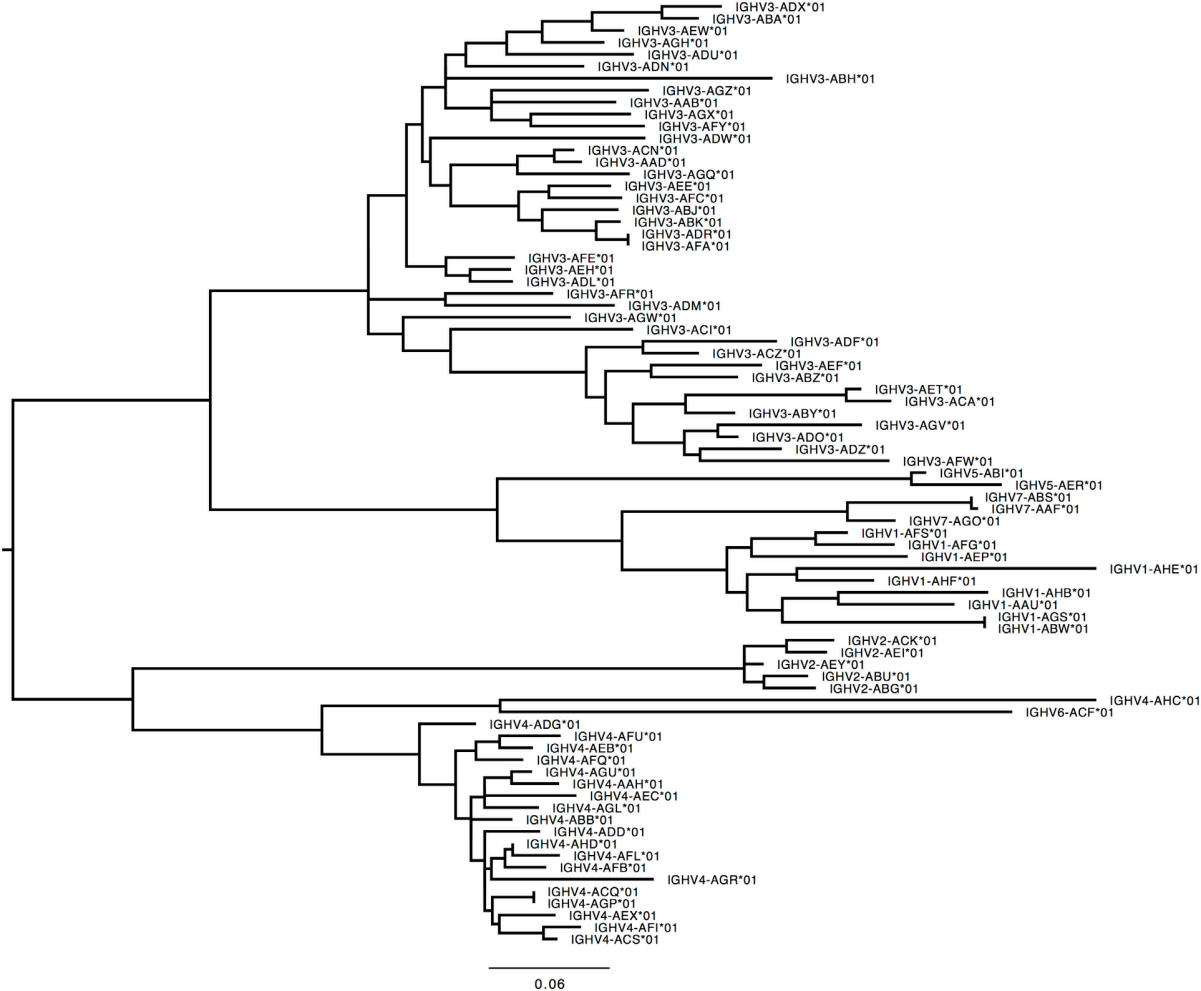
Maximum likelihood phylogenetic tree of the rhesus IGHV functional genes, based on the final immunoglobulin allele library.

276 Functional/ORF IGHV genes were found in M1–M9, including 142 unique functional genes and 5 unique ORFs (Table S5A in Supplementary Material). The largest number of genes/alleles was found in the IGHV3 family (Figure 4). The final IGHV allele library, comprising genes from the reference and supplementary assemblies, contains 72 functional genes with 186 allelic forms belonging to 7 families (Table 1), with the IGHV3 family being the largest (38 IGHV genes, 112 alleles). In addition, 12 alleles were found for 7 functional genes that were identified on contigs that could not be placed (Table 1). 4 IGHV3 ORFs (Table 1) were identified in M1–M9, all of which were absent from the reference IGH locus. Similarity of the genes within each family was highly variable with maximum variation in IGHV3 family.

**Table 1:** List of all functional and ORF IGHV genes and alleles in the IGHV allele library.

IGHV family	F genes (alleles)	Open reading frame genes (alleles)	F genes (alleles)^a^
IGHV1	5 + 3 (22)	–	1 (3)
IGHV2	5 (9)	–	1 (3)
IGHV3	35 + 3 (112)	0 + 4 (5)	–
IGHV4	13 + 2 (25)	–	5 (6)
IGHV5	2 (8)	–	–
IGHV6	1 (3)	–	–
IGHV7	3 (7)	–	–

*The number of genes in the table is represented as number of genes found in the high quality immunoglobulin (Ig) sequence (including sister scaffolds) + number of genes added from Ig-baiting sequencing. The ^a^ indicates that the location of the scaffold on the IGH locus is unknown*.

#### *IGHD* 

198 Functional IGHD genes were found in the nine macaques, including 47 unique functional IGHD genes. The IGHD allele library contains the 39 functional genes found in the reference assembly plus 10 alleles from the supplementary assemblies.

#### *IGHJ* 

56 IGHJ genes were found in the nine macaques including 9 unique IGHJ genes and 11 alleles. Two alleles were identified for the IGHJ1 and IGHJ3P genes. No IGHJ genes were identified that were not found in the reference assembly. In no monkeys were two copies of IGHJ5 found, although the reference does contain two copies, as noted above. With the exception of M1, the single copy of IGHJ5 was functional. For M1, the entire IGHJ cluster was assembled onto one contig, containing eight of the nine IGHJ found in the reference assembly (4 functional) and one IGHJ5P pseudogene. The complete IGHJ loci found in M0 and M1 were approximately 99.4% identical (Figure S2 in Supplementary Material) apart from a 402 bp indel.

#### *IGHC* 

Owing to low coverage, constant region genes were assembled into multiple contigs, typically distributing exons of the same gene into different contigs. This circumstance makes it impossible to unambiguously determine allelism. Nevertheless, the exons found on each of the supplementary assemblies were identical with or very similar to exons on each of the other supplementary assemblies and to exons on the reference assembly (Table S5B in Supplementary Material). While the IGHD genes were identical in all ten macaques, the other IGHC genes (IGHA, IGHE, IGHM, IGHG) were highly diverse between macaques (Table 2).

**Table 2:** List of IGHC alleles (functional, non-functional) in the IGHC allele library.

IGHC gene	CH1	CH2/H-CH2	CH3/CH3-CHS	CH4/CH4-CHS	H1	M1	M2
IGHA	4	3	3	–	–	1	–
IGHD	1	1	1	–	1	1	–
IGHE	4	1	2	5	–	2	–
IGHEP	1	2	2	3	–	–	–
IGHM	4	3	2	5	–	1	–
IGHG1	1	3	3	–	–	1	1
IGHG2	1	1	2	–	–	1	1
IGHG3	3	2	3	–	1	1^a^	1
IGHG4	3	1	3	–	–	–	1^a^

*The ^a^ indicates that two copies of the gene are found in the macaque genome*.

#### *IGKV* 

359 Functional/ORF IGKV genes were found in M1–M9, including 177 unique, functional genes, and 6 unique ORFs (Table S6A in Supplementary Material, Figure 5). The IGKV allele library contains a total of 70 functional genes with 214 alleles. Three IGKV ORF’s with 6 alleles were identified (Table 3).

**Figure 5:**
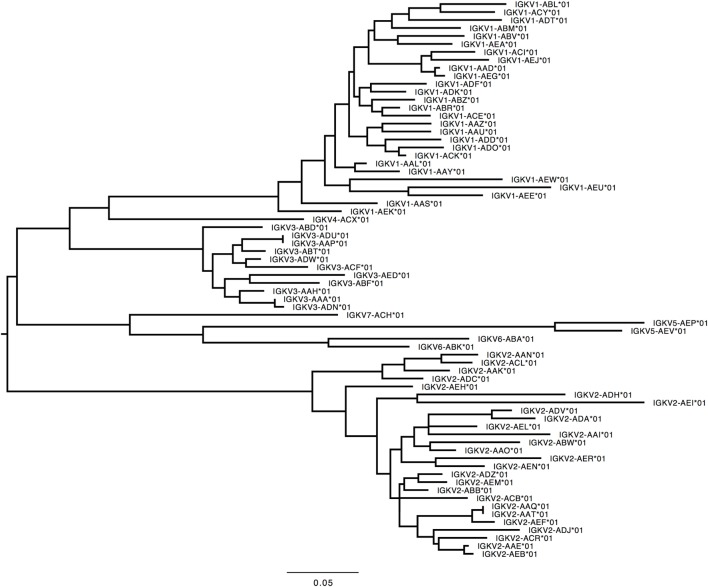
Maximum likelihood phylogenetic tree of the rhesus IGKV functional genes, based on the final immunoglobulin allele library.

**Table 3:** List of all functional and open reading frame (ORF) IGKV genes and alleles in the IGKV allele library.

IGKV family	F genes (alleles)	ORF genes (alleles)
IGKV1	25 + 2 (80)	0 + 1 (1)
IGKV2	25 + 1 (88)	0 + 2 (5)
IGKV3	11 (33)	–
IGKV4	1 (1)	–
IGKV5	0 + 2 (5)	–
IGKV6	2 (5)	–
IGKV7	1 (2)	–

*The number of genes in the table is represented as number of genes found in the high quality immunoglobulin (Ig) sequence (including sister scaffolds) + number of genes added from Ig-baiting sequencing*.

#### *IGKJ* 

The IGKJ cluster was assembled into one contig each for each of the nine macaques. Each such cluster contained four functional genes and one pseudogene (IGKJ5), with similarity among alleles uniformly above 99%. The IGKJ clusters in two monkeys (M2, M8) are identical to that of the reference.

#### *IGKC* 

The single IGKC was found in all animals in six unique allelic forms. The assembly containing IGKC in four monkeys (M2, M4, M6, and M9) are identical to that of the reference.

#### *IGLV* 

**Figure 6:**
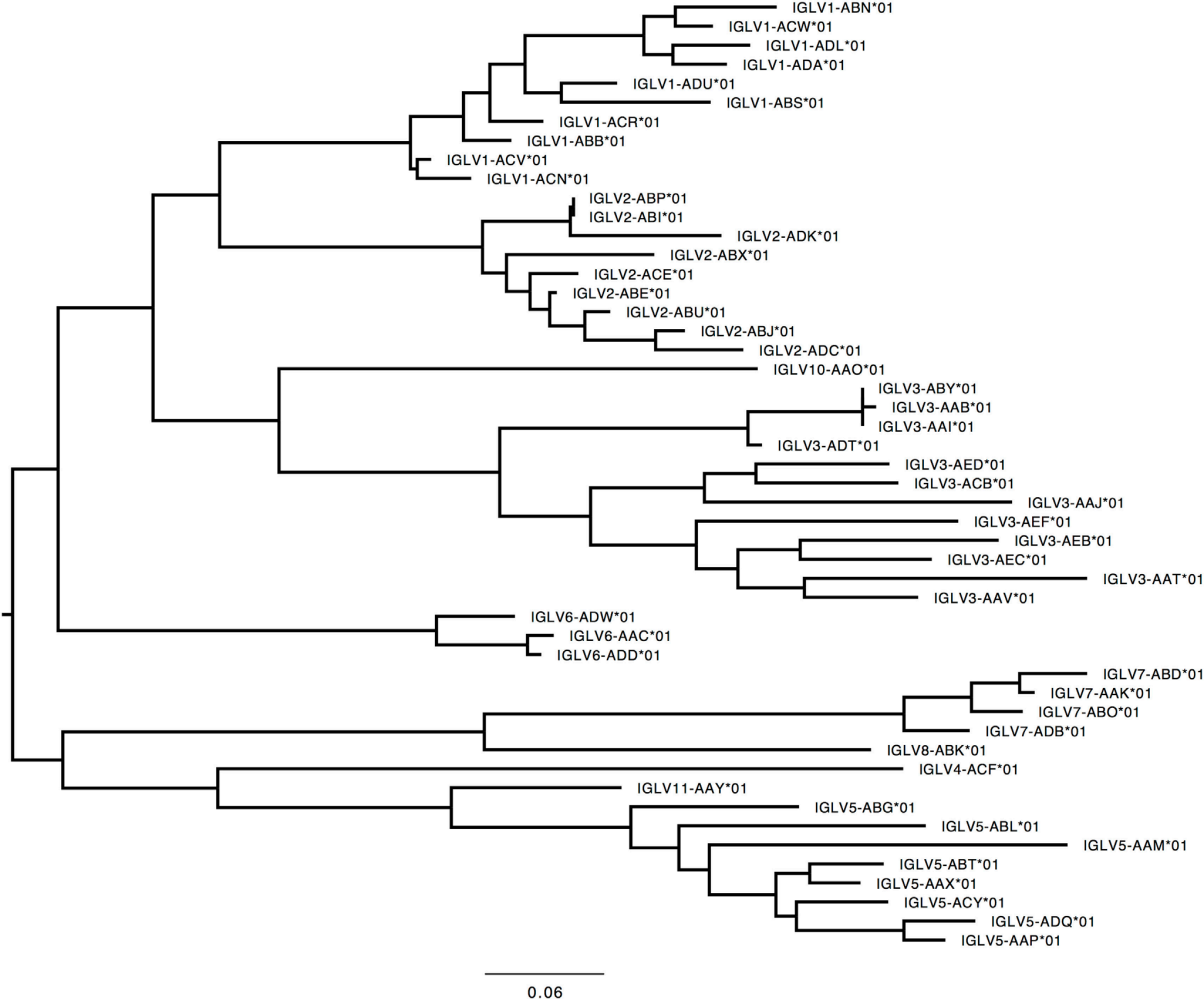
Maximum likelihood phylogenetic tree of the rhesus IGLV functional genes, based on the final immunoglobulin allele library.

291 genes were found, including 125 unique functional genes and 5 ORFs (Table S6B in Supplementary Material). The IGLV allele library contains 50 functional genes with 137 alleles and 2 ORFs with 5 alleles (Table 4, Figure 6). Similarity of genes within each family varied by family.

**Table 4:** List of all functional and open reading frame (ORF) IGLV genes and alleles in the IGLV allele library.

IGLV family	F genes (alleles)	ORF genes (alleles)
IGLV1	10 (35)	–
IGLV2	9 (18)	–
IGLV3	8 + 4 (38)	–
IGLV4	1 (4)	–
IGLV5	8 (19)	–
IGLV6	3 (11)	–
IGLV7	4 (9)	1 + 0 (2)
IGLV8	1 (3)	0 + 1 (3)
IGLV9	–	–
IGLV10	1 (1)	–
IGLV11	1 (2)	–

*The number of genes in the table is represented as number of genes found in the high quality immunoglobulin (Ig) sequence (including sister scaffolds) + number of genes added from Ig-baiting sequencing*.

#### *IGLJ* 

41 genes were found including 10 unique functional genes. IGL4 and IGL5 had mutated recombination signal sequences and were NF. The IGLJ allele library contains 7 IGLJ with 11 alleles.

#### *IGLC* 

Very few IGLC genes were identified in M1–M9. 16 genes were found, including 12 unique sequences. Of the 12 alleles identified, only 2 (IGLC2/IGLC3 and IGLC4P) of these were identical to the IGLC genes identified in the reference assembly. The final IGLC allele library consisted of 7 IGLJ genes (17 alleles).

### Interspecies Ig Diversity: Comparison of Human and Rhesus Macaque Ig Genes

#### *IGHV* 

**Figure 7:**
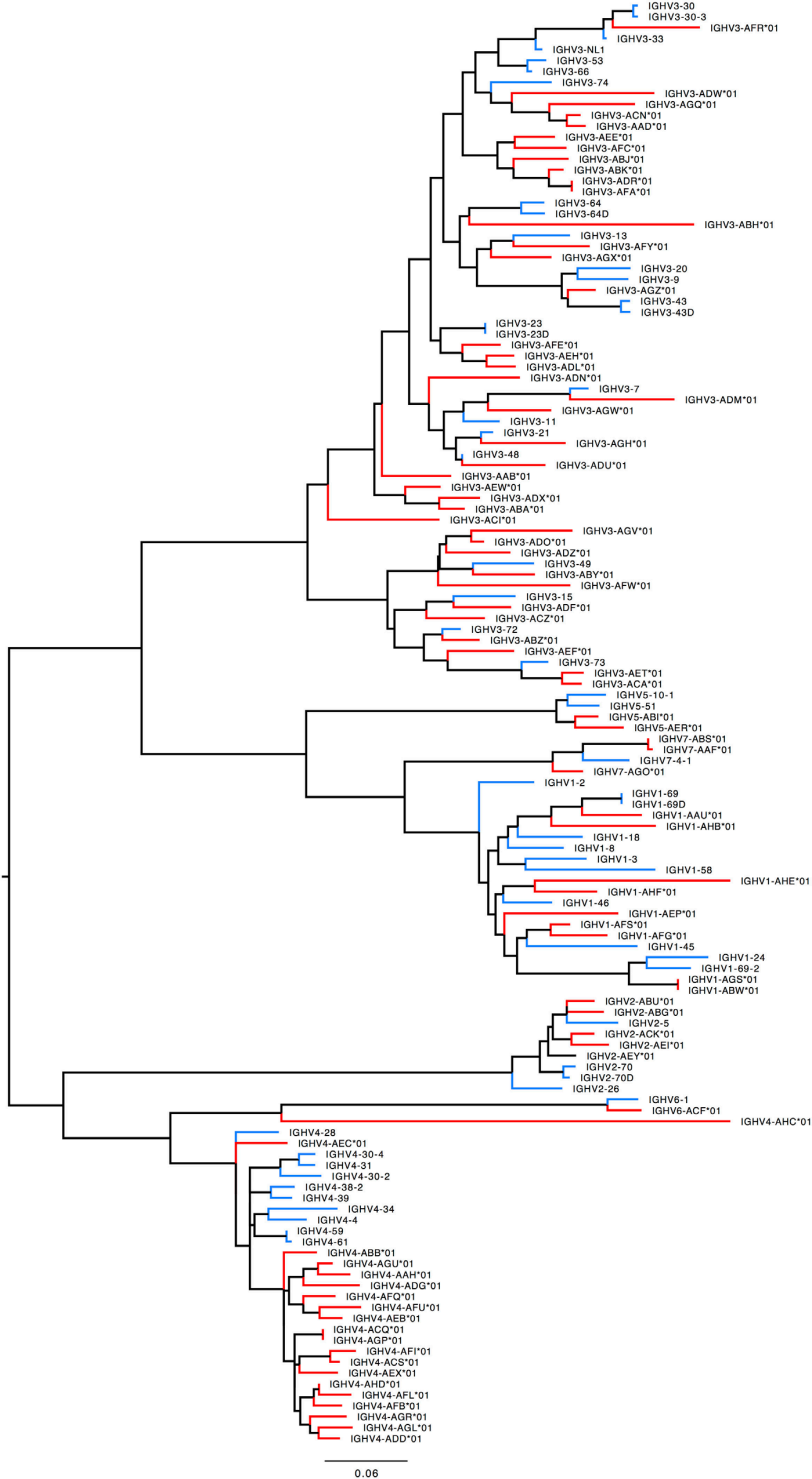
Maximum likelihood phylogenetic tree of the human and rhesus IGHV functional genes. Human immunoglobulin (Ig) V genes are represented in blue and macaque Ig V genes in red.

Humans typically carry 123–129 total IGHV genes in the IGH locus per haploid genome, including 38–46 functional genes classified into 7 families (32). Table S7A in Supplementary Material lists genes and alleles found in each of the families in humans and macaques, respectively. The number of functional genes in macaques includes genes found both on sister scaffolds and scaffolds that could not be placed. Both humans and macaques have similar numbers of genes, with the macaque IGHV3 family larger in comparison to humans. The allelic diversity exhibited in these ten macaques is on a similar scale to the allelic diversity of the human IGHV genes contained in total in the IMGT database. Genes in the IGHV1, IGHV2, IGHV3, IGHV6, and IGHV7 families are phylogenetically interdigitated, suggesting intra-family divergence prior to the macaque-human divergence, genes in the IGHV4 and IGHV5 families segregate by species, suggesting post-speciation homogenization in these families (Figure 7).

#### *IGHD* 

Humans typically have 23 functional IGHD genes belonging to seven families and an additional 14 ORFs (32). We identified 39 IGHD genes with 49 alleles in macaques in six families (Figure S3A in Supplementary Material). Macaques thus have a substantially increased IGHD repertoire.

#### *IGHJ* 

In humans, 17 alleles representing nine IGHJ genes (six functional) have been found. Macaques also have nine IGHJ genes but apparently do not have a functional IGHJ2. Some macaques may carry two copies of IGHJ5. Most macaque IGHJ genes have distinct human orthologs (Figure S3B in Supplementary Material).

#### *IGHC* 

57 IGHC alleles representing 12 IGHC genes (2 IGHA, IGHD, IGHE, 2 IGHEP, 4 IGHG, IGHGP, and IGHM) have been identified in humans. Macaques appear to have only eight IGHC genes, lacking IGHGP, one IGHEP and one *IGHA*. IGHC genes have greater numbers of alleles than humans (Table S7B in Supplementary Material). Pairwise identity between macaque and human IGHC exons is uniformly greater than 80% (Table S7C in Supplementary Material). All IGHG genes segregate by species (Figure S3C in Supplementary Material).

#### *IGKV* 

**Figure 8:**
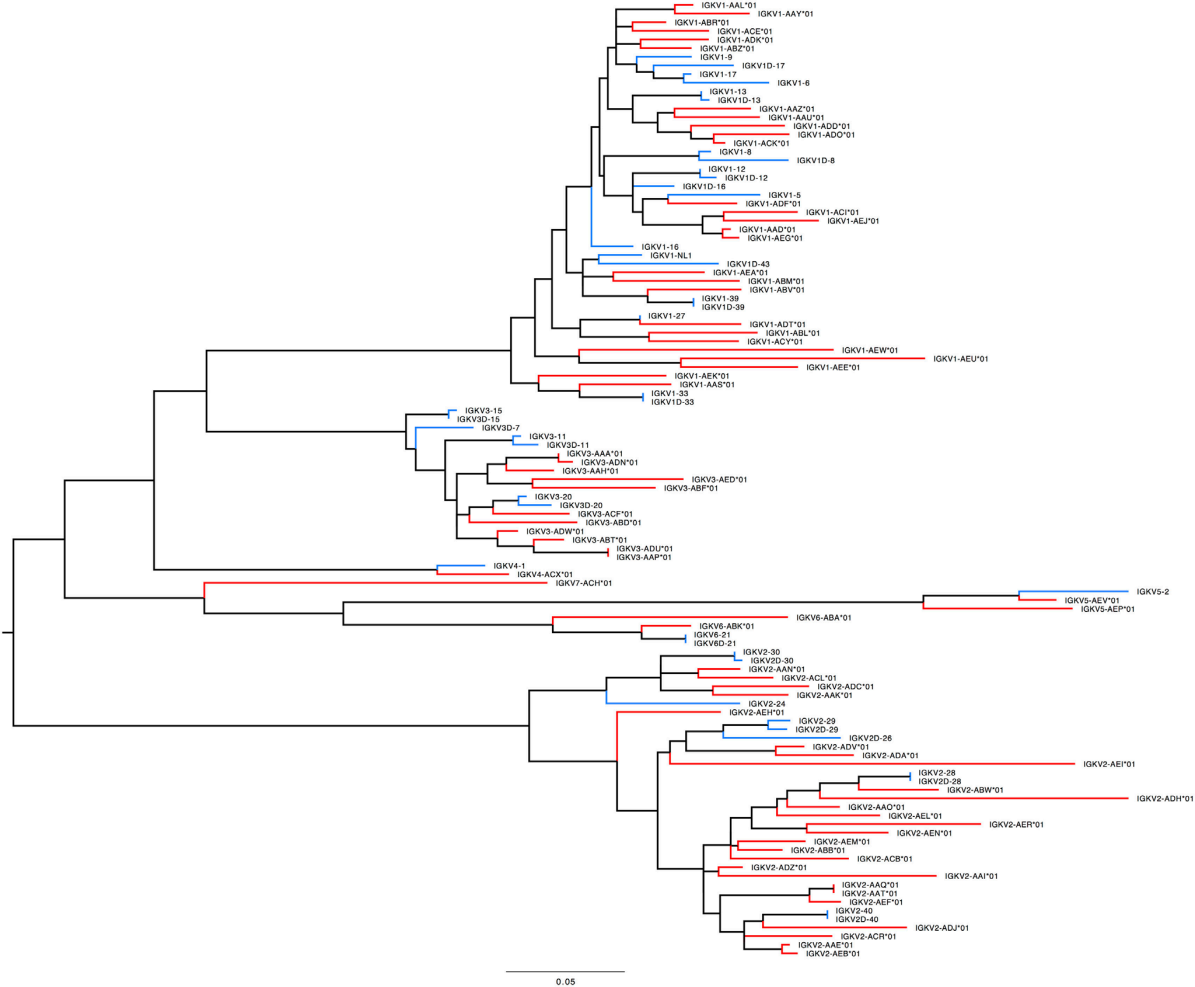
Maximum likelihood phylogenetic tree of the human and rhesus IGKV functional genes. Human immunoglobulin (Ig) V genes are represented in blue and macaque Ig V genes in red.

In humans, 41 functional IGKV genes with 67 alleles representing 6 families and 10 ORFs (16 alleles) have been identified. Table S7D in Supplementary Material lists genes and alleles found in each of the families in humans and macaques, respectively. The number of functional genes in macaques includes genes found on the sister scaffold. With the exception of the IGKV2 family, both humans and macaques have similar number of genes. The macaque IGKV genes are also more polymorphic in comparison to the human IGKV genes. In addition, unlike macaques, humans lack functional genes in the IGKV7 family. A phylogenetic tree (Figure 8) of macaque and human IGKV genes indicates that genes all genes in the IGKV families are interdigitated. Similar to macaques, humans have functional IGKV genes in the forward and the reverse orientation. Humans have 3 alternating blocks of IGKV genes—with 2 large blocks of IGKV genes—representing a duplication event in the human IGKV locus. While macaques have 6 alternating blocks of IGKV genes in the forward and reverse orientation, the 2 large blocks of genes in the forward and reverse orientation do not represent duplication events.

#### *IGKJ* 

Humans have 5 functional IGKJ genes (9 alleles). A phylogenetic tree (Figure S4 in Supplementary Material) of human and macaque IGKJ genes indicated that all macaque IGHJ genes have distinct human orthologs.

#### *IGKC* 

Similar to macaques, humans have 1 IGKC gene (5 alleles). 6 alleles of the IGKC gene were found in 10 macaques, indicating that the IGKC gene is highly diverse in macaques, in comparison to humans. The pairwise identity between human and macaque IGKC is 91.6%.

#### *IGLV* 

**Figure 9:**
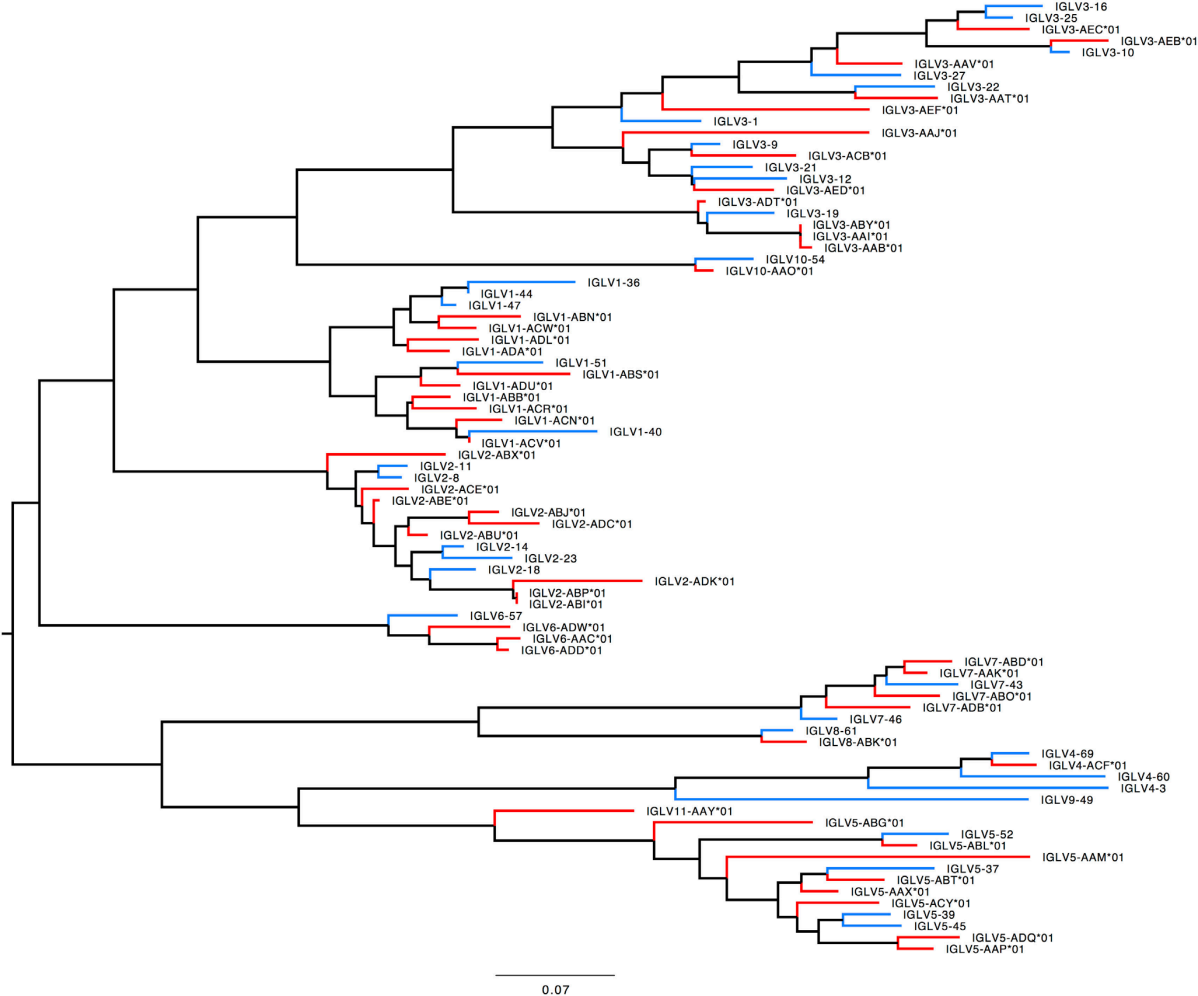
Maximum likelihood phylogenetic tree of the human and rhesus IGLV functional genes. Human immunoglobulin (Ig) V genes are represented in blue and macaque Ig V genes in red.

In humans, 33 functional IGLV genes represented by 71 alleles in 10 families, plus seven ORFs with 11 alleles have been identified. The IGLV11 family exists, but no functional genes belonging to that family have been observed in humans. In rhesus macaque, functional members of IGLV9 have not been observed. Most IGLV families—IGLV1 to IGLV7—are larger and more diverse in macaques (Table S7E in Supplementary Material; Figure 9).

#### *IGLJ* 

Five functional genes (with IGLJ2 and IGLJ3 identical) and two NF genes have been found in humans—we identified five functional genes in the macaque (Figure S5A in Supplementary Material).

#### *IGLC* 

In humans, it appears to be typical to carry five functional genes and two NF genes per haploid locus (though there are reports of NF versions of IGLC6 (32), with 21 alleles). We find the same organization in our macaques. Interestingly, the functional genes segregate by species while the NF genes segregate into ortholog pairs (Figure S5B in Supplementary Material).

### Sequence Diversity

We computed the average pairwise distance over unique genes in the macaque and human collections. For a gene to be included in the analysis, it had to have both canonical cysteines, be in-frame, contain no stop codons, be full length, and contain no ambiguous nucleotides. For human genes, we had 201 such genes. For macaque genes, we had 114. For all families but IGHV4, the mean pairwise distance (MPD) is substantially larger in the macaque genes (Table 5).

**Table 5:** Comparison of the mean pairwise distance (MPD) in individual IGHV gene families in human and macaque.

Family	Human		Macaque		ΔM/M (%)
	Size	MPD (%)	Size	MPD (%)	
IGHV1	31	8.7	11	11.0	26.5
IGHV2	14	4.5	6	5.3	16.5
IGHV3	98	9.5	69	12.7	33
IGHV4	45	6.2	13	5.3	−15.2
IGHV5	7	3.2	6	5.2	59.6
IGHV6	2	0.3	2	0.3	0.0
IGHV7	4	0.6	7	5.0	786

*Size is the total number of genes in the family. ΔM/M = (mMPD − hMPD)/hMPD*.

## Discussion

We have assembled and characterized in detail the Ig loci (IGH, IGK, and IGL) of the rhesus macaque in a single high-coverage whole-genome assembly, and in targeted sequencing and assembly of nine additional individuals, providing information on allelic polymorphism.

### Within-Macaque Diversity

The assembly and ordering of our scaffolds into two distinct overlapping loci at IGHV and IGKV suggest substantial polymorphism between homologous chromosomes. Although the methods used were not devised to resolve homologous chromosomes in diploid genomes (so are likely to have produced some errors in the partially diploid assembly), other results provide additional evidence in favor of this interpretation. Very long scaffolds were obtained in regions where little polymorphism is expected. These include a single 1.4 Mb-long scaffold for IGH that appears to be complete for IGHC, IGHJ, IGHD, and part of IGHV. Beyond this point, the scaffolds are much smaller and only partially ordered. Similarly, the largest scaffold for IGL contains all of the constant-region and J-genes as well as several IGLV. The IGK locus contains only a single constant-region gene; that together with all of the IGKJ is on a single locus, though it is not the largest scaffold.

Furthermore, the ordering and annotation of the smaller scaffolds for both IGH and IGK suggest copy-number variation between the homologous chromosomes bearing these loci. If true, this would explain the greatly increased difficulty of assembling these regions relative to those more highly conserved. Copy-number differences among haplotypes have been observed now in precise analyses of human Ig loci made possible by the sequencing of haploid cellular clones from hydatidiform moles (33).

### Among-Macaque Diversity

The MPD within IGHV families is substantially larger in macaques than in humans (Table 5). The inferred V and C region genes are highly polymorphic among the ten macaques, while the J and D genes are conserved. The exceptional diversity among Ig V and C genes is unlikely to be due to sequencing or assembly error since (1) the Ig C, J, and where relevant, the Ig D genes, are assembled onto a single scaffold in the reference genome; (2) the J and D genes are identical across all ten macaques. Furthermore, this diversity is consistent with the nature and extent of diversity found at the macaque MHC (34).

The use of bait-and-sequence appears to have had an impact on our results. Human probes were used as baits; where human and macaque sequences share significant sequence identity, we assembled high coverage macaque Ig counterparts. For example, in the case of IGKJ genes, three of the five macaque genes were identical to humans resulting in the complete assembly of IGKJ in all nine macaques. In contrast, when macaque and human genes segregated by species on a phylogenetic tree, we assembled low- or no-coverage contigs (IGHC genes, IGLC genes, IGHV4, IGHV5 genes) Ig genes in the nine assemblies. Given the design of the molecular baits based on human sequences, we believe that the true nature of Ig diversity is underrepresented in some of the Ig gene families that segregate by species.

### Interspecies Diversity between Humans and Macaques

Relative to humans, several V gene families in all the three Ig loci were expanded in macaques. In addition, the diversity of the macaque Ig V genes was several times higher than observed in humans. For example, we identified 197 alleles for all functional IGHV genes in 10 macaques, while only 273 functional IGHV alleles have been reported in the human IMGT database, a repository of all human Ig sequences that have been studied to date. We identified similar levels of allelic diversity in all three macaque Ig loci (214 alleles in macaque IGKV, 140 alleles in macaque IGLV), which is strikingly different than reports in humans (67 alleles in human IGKV, 71 alleles in human IGLV). It is important to keep in mind that while the diversity of the Ig loci is a collection of all humans studied to date, no study has been undertaken to specifically understand the diversity of the human Ig loci is such detail.

### Orthology and Gene Conversion

The Ig gene families exhibit distinctly different patterns of phylogenetic segregation between macaque and human. While human and macaque genes interdigitated for many genes in most families, genes in some families segregated by species (IGH: IGHV4, IGHV5, IGHG; IGL: IGLJ2, IGLJ3, IGLC1-3, IGLC6, IGLC7). One possible explanation for this phenomenon is that these latter families were generated post-speciation by duplication of different effective founder genes in the two lineages with the concomitant loss of other family members. More likely is the hypothesis that the families were fully populated at species divergence and homogenized afterward. If true, the likely mechanism underlying this phenomenon would be gene conversion. This possibility is intriguing because gene conversion can contribute to diversification, as has been documented for the HLA class II region (35), as well as to homogenization (36). Evidence for sustained gene conversion activity in highly polymorphic parts of the human genome has been observed by others (37).

Circumstantial evidence for this hypothesis includes our observation that human Ig genes from families that segregate by species share physical proximity on the chromosome with genetic proximity in the phylogenetic tree. The members of these gene pairs are closest to each other both physically and by sequence similarity: IGHV4-38/IGHV4-39, IGHV4-59/IGHV4-61, IGHV4-30/IGHV4-31; IGLJ2 and IGLJ3; IGLC1-3, IGLC6 and IGLC7. The two NF IGLC genes (IGLC4P and IGLC5P) do not segregate by species (Figure S5B in Supplementary Material).

In contrast, in the gene families that interdigitate rather than segregate, we have the following. IGLV3: no dual-adjacency pairs are found. IGHV3: there are two dual-adjacency pairs: IGHV3-30/IGHV3-33 and IGHV3-72/IGHV3-73 (Figures S6A,B in Supplementary Material). The former pair segregates by species (Figure S6A).

In addition, we identified signatures attributable to gene conversion in macaque Ig V genes. Such signatures are defined in quartets of genes that have two clusters of sequence polymorphisms separated from each other in the gene (call them A, a; and B, b). The signature is present if there are four genes that together contain all four combinations (Figure S7 in Supplementary Material). By manual inspection of the macaque Ig V genes, we found three such cases (Table S8 in Supplementary Material). In sum, it appears that, consistent with reports on other immune loci in primates and humans (37, 38), the macaque Ig locus is rapidly evolving with gene conversion playing a key role in generating diversity while maintaining functionality.

### Applications

One important application of the information obtained in this study is in vaccine development using macaque models. Human IGHV1-69 and IGHV1-2 alleles appear to be favored for eliciting effective BNAbs against influenza and HIV-1, respectively (9, 39). A preliminary version of our germline gene library was used to study Ig responses in macaques immunized with the HIV-1 envelope and adjuvants (40), concluding that the IGHV genes, IGHV4A*01, IGHV4D*01, and IGHV3J*01 were specifically associated with neutralizing responses following SHIV_AD8_ infection and IGHV3Q*01 in protein/adjuvant immunization. This is particularly important as the IGHV3Q*01 is very similar to the human IGHV gene used by the CAP256-VRC26 family of bnAbs (40) raising the odds that similar antibodies might be elicited in the macaque. Many of the Ig germline genes do not have true orthologs between human and macaque, but most of them share 90% or greater identity with one or more genes in the other species. The macaque Ig germline V-genes with similarity to the human germline genes of greatest interest for bnAbs are tallied in Table 6.

**Table 6:** Heavy and light chain V gene usage in human neutralizing and broadly neutralizing bodies with the corresponding closest macaque V genes.

Viral epitope	Antibody clonal Family	Human heavy chain V gene	Human light chain V(k/λ) gene	closest Macaque heavy chain V gene (percent identity)	closest Macaque light chain V gene (percent identity)
MPER of gp41	2F5	2–5	κ1–13	2-ABU (94.35)	κ1-AAZ (92.33)
	4E10	1–69	κ3–20	1-AAU (93.24)	κ3-ADW (94.48)
	M66.6	5–51	κ1–39	5-ABI (94.93)	κ1-ABV (94.42)
	CAP206–CH12	1–69	κ3–20	1-AAU (93.24)	κ3-ADW (94.48)
	10E8	3–15	λ3–19	3-ADF (91.72)	**λ**3-ADT (95.17)

V1V2-glycan	PG9, PG16	3–33	λ2–14	3-AFR (93.24)	λ2-ABU (94.27)
	CH01–04	3–20	κ3–20	3-AGZ (93.24)	κ3-ADW (94.48)
	PGT 141–145	1–8	κ2–28	1-AHB (87.24)	κ2-ABW (94.70)

Outer domain glycan	2G12	3–21	κ1–5	3-AGH (93.52)	κ1-ADF (93.37)
	PGT121–123	4–59	λ3–21	4-AEC (92.22)	λ3-AED (91.78)
	PGT125–131	4–39	λ2–8	4-AEC (90.39)	λ2-ACE (94.27)
	PGT135–137	4–39	κ3–15	4-AEC (90.39)	κ3-AAH (92.73)

CD4 binding-site	b12	1–3	κ3–20	1-AHF (86.82)	κ3-ADW (94.48)
	HJ16	3–30	κ4–1	3-AFR (92.22)	κ4-ACX (95.08)
	CH103–106	4–59	λ3–1	4-AEC (92.22)	λ3-AEF (84.53)
	VRC01–03	1–2	κ3–20	1-AFS (91.21)	κ3-ADW (94.48)
	VRC-PG04, 04b	1–2	κ3–20	1-AFS (91.21)	κ3-ADW (94.48)
	VRC-CH30–34	1–2	κ1–33	1-AFS (91.21)	κ1-AAS (93.72)
	3BNC117, 3BNC60	1–2	κ1–33	1-AFS (91.21)	κ1-AAS (93.72)
	NIH45–46	1–2	κ3–20	1-AFS (91.21)	κ3-ADW (94.48)
	12A12, 12A21	1–2	κ1–33	1-AFS (91.21)	κ1-AAS (93.72)
	8ANC131, 134	1–46	κ3–20	1-AHF (89.18)	κ3-ADW (94.48)
	CH235	1–46	κ3–15	1-AHF (89.18)	κ3-AAH (92.73)
	1NC9, 1B2530	1–46	λ1–47	1-AHF (89.18)	λ1-ACW (93.92)

gp120-gp41	35O22	1–18	λ2–14	1-AHB (86.86)	λ2-ABU (94.27)
	PGT151	3–30	κ2D–29	3-AFR (92.22)	κ2-ADV (92.38)
	8ANC195	1–69	κ1–5	1-AAU (93.24)	κ1-ADF (93.37)

The database of macaque germline Ig genes resulting from this work is the largest such collection currently available. It is also thoroughly annotated and mapped to the genomic context in which they were found. While we are very pleased to provide this resource to the community, we must point out that our findings provide a strong argument against the naïve use of germline databases for the analysis of IgVRG repertoire sequencing. In the face of such extensive diversity, the likelihood that all the germline Ig genes of the animal subject are to be found in the database is small. Furthermore, the unlimited collection of germline genes into an ever-growing database is not a workable solution. The more germline genes, the greater the chance of false-positive identifications. It is evident that the inference of germline genes directly from mature, recombined IgVRG is of great interest. Initial work in this area includes that of the Collins group (41) and the Kleinstein group (42), and the more recent methods from Corcoran et al. (43). With these very promising beginnings, a complete, practical, and statistically sound method will be developed. Even then, there will remain a great deal to learn about adaptive immunity from careful comparative genomic study of the antigen receptor loci.

Our results are consistent with those of several other studies showing that genetic diversity is substantially lower generally in humans compared with other primates (44). This conclusion has substantial consequences for the interpretation of immunological experiments with rhesus macaques. In studies of the humoral response, distinguishing somatic mutations from allelic polymorphisms is more difficult in macaques and will require a great deal more care and effort. It may be advisable to consider genetic diversity in the design of biomedical studies with macaques and other non-human primates. It will be interesting to learn whether the increased genetic diversity of the Ig loci is accompanied by functional diversity of the humoral response, or simply provides alternative parallel paths to the same effective solutions. Beyond biomedicine, these studies contribute further evidence that the modes of evolution of the Ig loci are active, that is, under the regulatory influence of host factors, and worthy of substantial further research. Furthermore, the rhesus macaque may be the ideal model for such investigations.

### A Note on Nomenclature

The convention for naming Ig germline genes is to combine the family designation with the order of the gene on the chromosome. Thus IGHV2-5 is a member of IGHV family five and is the 5th closest functional IGHV gene to the IGHJ cluster. It has been shown now that the extent of copy number variation in human Ig loci is substantially greater than was anticipated at the time this convention was adopted (3). The work we report here suggests similar or greater copy-number variation in the macaque Ig loci. Therefore, we are not adopting the standard naming convention. Instead we use the family designation—at this time, we have no reason to believe that this designation will be problematic—but use a random three-letter combination to designate genes. Thus in the macaque we have, e.g., IGHV2-ABG.

## Ethics Statement

This study was carried out in accordance with National Institute of Health guidelines; all animal procedures were approved by the Vaccine Research Center’s Institutional Animal Care and Use Committee.

## Author Contributions

AR designed the assembly procedure, carried out the assembly, performed the analyses, and wrote the paper; SD designed the sequencing baits and collected and processed the DNA sequence data; AH developed software for supplemental analyses; GO procured and processed the tissue from the reference macaque; AR performed genomic sequencing; JF designed the sequencing baits; AT managed the sequencing data; GT oversaw tissue collection and edited the paper; BH helped develop the project and edited the paper; DD conceived the project and edited the paper; TK conceived the project, assisted with data analysis, and wrote the paper.

## Conflict of Interest Statement

The authors declare that the research was conducted in the absence of any commercial or financial relationships that could be construed as a potential conflict of interest.
