# Supplementary material for: Structure and Diversity of the Rhesus Macaque Immunoglobulin Loci through Multiple De Novo Genome Assemblies
Source: Front Immunol. 2017 Oct 27;8:1407. doi: 10.3389/fimmu.2017.01407 (PMC5663730; doi:10.3389/fimmu.2017.01407)
Supplement: Supplementary file 1 [file presentation_1.pdf]

Structure and diversity of the rhesus macaque immunoglobulin loci through multiple *de novo* genome assemblies

Akshaya Ramesh[1], Sam Darko[2], Axin Hua[4], Glenn Overman[3], Amy Ransier[2], Ashley Trama[3], Georgia D. Tomaras[3], Barton F. Haynes[3], Daniel C. Douek[2,*], Thomas B. Kepler[4,5,*]

[1]Graduate Program in Genetics and Genomics, Boston University School of Medicine, Boston, MA, USA

[2]NIH Vaccine Research Center, National Institute of Allergy and Infectious Diseases, National Institutes of Health, Bethesda, MD, USA

[3]Duke Human Vaccine Institute, Duke University Medical Center, Durham, NC, USA.

[4]Department of Microbiology, Boston University School of Medicine, Boston, MA, USA

[5]Department of Mathematics and Statistics, Boston University, Boston MA, USA

*Joint senior authors

*** Correspondence:**
Daniel C. Douek

ddouek@mail.nih.gov

Thomas B. Kepler

tbkepler@bu.edu

1 Supplementary Data

Supplemental Spreadsheet 1: MUMmerv3.23 alignments of IGH contigs aligned to African Green monkey IGH locus. Legend: The highlighted region (yellow) indicates the coordinates that were taken into consideration to determine final IG order. One of the sister IGH contigs, scaffold 5775, had 2 conflicting coordinates (highlighted in red) when aligned to the African green monkey IGH locus. In both these cases, final contig order remains unaffected.

Supplemental Spreadsheet 2: MUMmerv3.23 alignments of IGK contigs aligned to African Green monkey IGK locus. Legend: The highlighted region (yellow) indicates the coordinates that were taken into consideration to determine final IG order.

Supplemental Spreadsheet 3: MUMmerv3.23 alignments of IGL contigs aligned to African Green monkey IGL locus. Legend: The highlighted region (yellow) indicates the coordinates that were taken into consideration to determine final IG order.

[S1] start of the alignment region in the reference sequence (Chlorocebus sabaeus Ig) [E1] end of the alignment region in the reference sequence (Chlorocebus sabaeus Ig)[S2] start of the alignment

region in the query sequence [E2] end of the alignment region in the query sequence [LEN 1] length of the alignment region in the reference sequence [LEN 2] length of the alignment region in the query sequence [% IDY]percent identity of the alignment [COV R] percent alignment coverage in the reference sequence (Chlorocebus sabaeus Ig) [COV Q] percent alignment coverage in the query sequence [Q-ID] ID of Query sequence

2 Supplementary Figures and Tables

2.1 Supplementary Figures

Fig. S1: Maximum likelihood phylogenetic tree of rhesus (A) IGHV (B) IGKV (C) IGLV functional genes from the high quality macaque. The Ig V gene families are represented by different colors (see legend).

1A

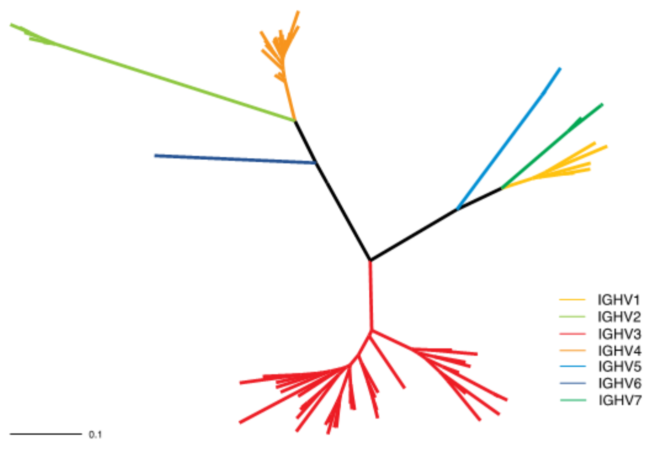

1B

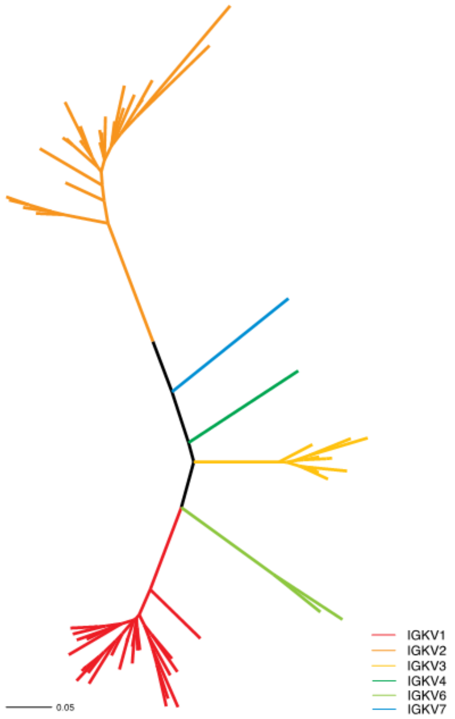
IGKV1
IGKV2
IGKV3
IGKV4
IGKV6
IGKV7
0.05

1C

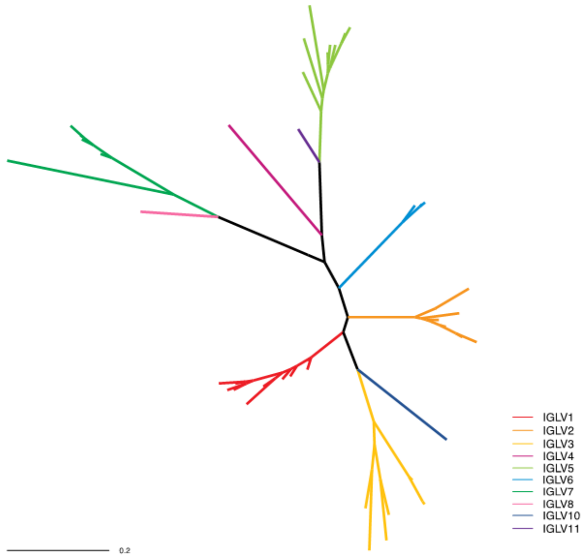
IGLV1
IGLV2
IGLV3
IGLV4
IGLV5
IGLV6
IGLV7
IGLV8
IGLV10
IGLV11
0.2

Fig. S2: Dot plot between IGHJ cluster of high quality Ig sequence macaque and Macaque 1 (Ig-baiting).

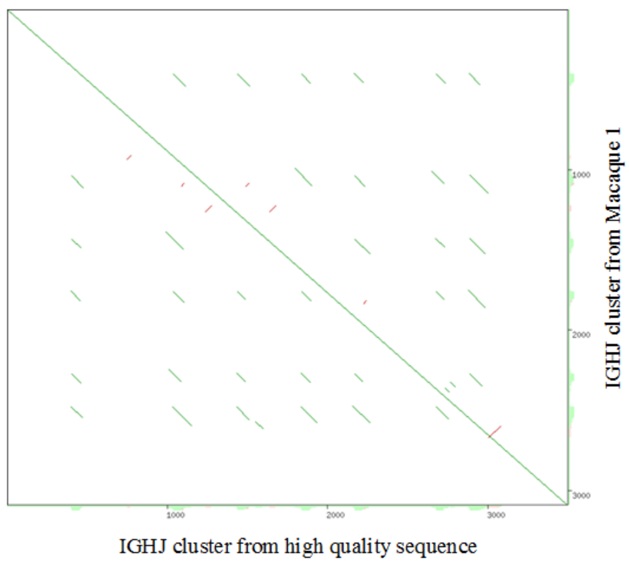

Fig. S3: Maximum likelihood phylogenetic tree of the human and rhesus (A) IGHD (B) IGHJ (C) IGHG genes. Human Ig genes are represented in blue and macaque Ig genes in red.

3A

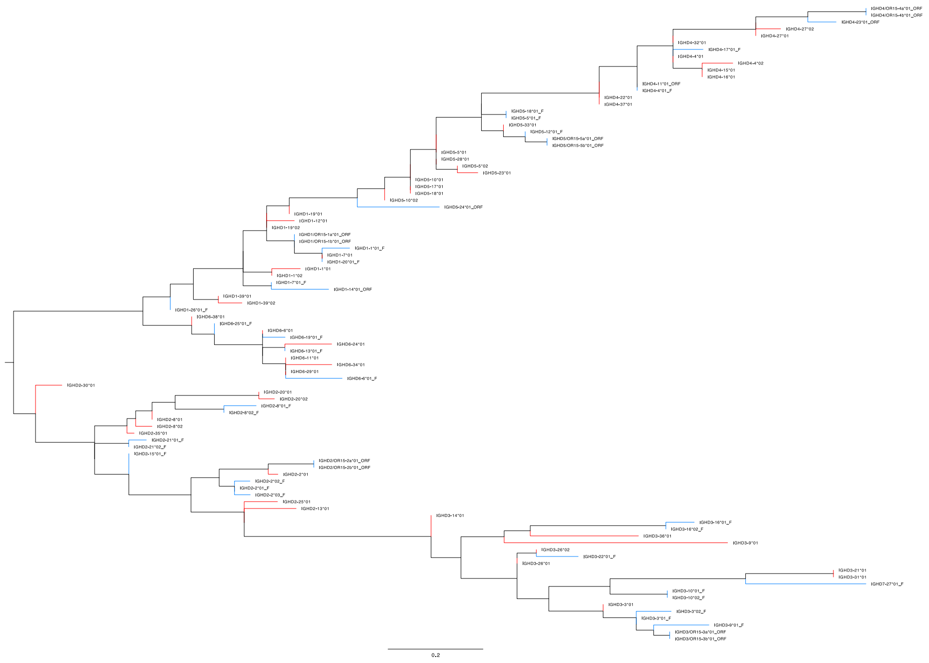

3B

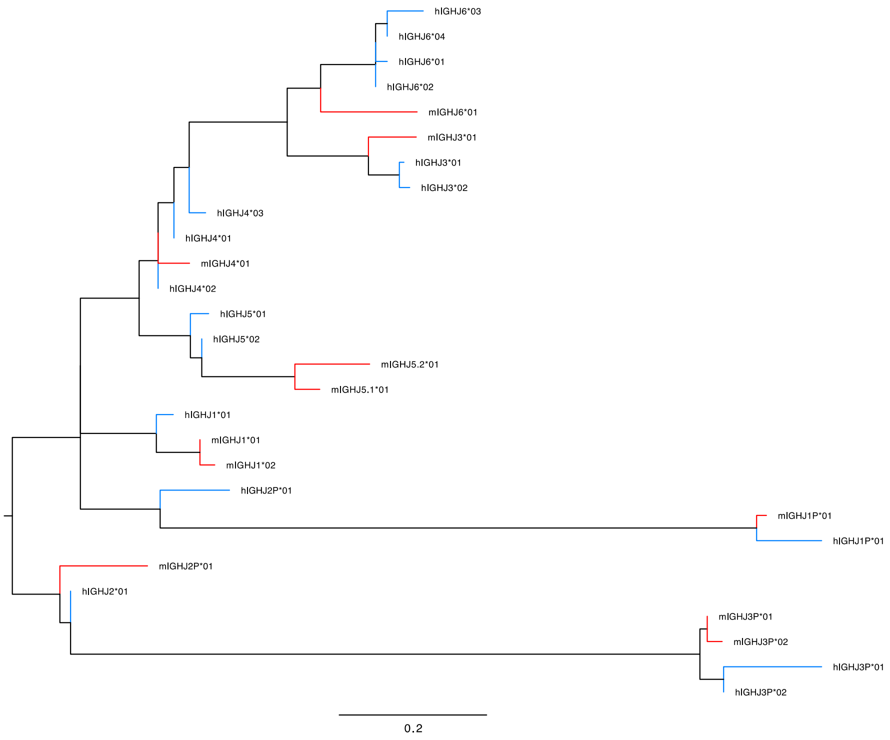
hIGHJ6*03
hIGHJ6*04
hIGHJ6*01
hIGHJ6*02
mIGHJ6*01
mIGHJ3*01
hIGHJ3*01
hIGHJ3*02
hIGHJ4*03
hIGHJ4*01
mIGHJ4*01
hIGHJ4*02
hIGHJ5*01
hIGHJ5*02
mIGHJ5.2*01
mIGHJ5.1*01
hIGHJ1*01
mIGHJ1*01
mIGHJ1*02
hIGHJ2P*01
mIGHJ1P*01
hIGHJ1P*01
mIGHJ2P*01
hIGHJ2*01
mIGHJ3P*01
mIGHJ3P*02
hIGHJ3P*01
hIGHJ3P*02
0.2

3C

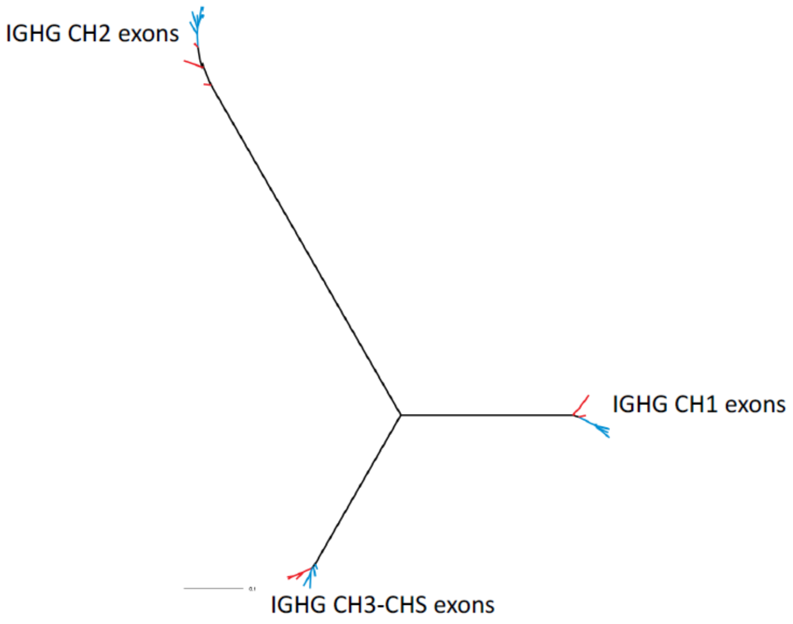
IGHG CH2 exons
IGHG CH1 exons
IGHG CH3-CHS exons

Fig. S4: Maximum likelihood phylogenetic tree of human and macaque IGKJ genes. Human Ig genes are represented in blue and macaque Ig genes in red.

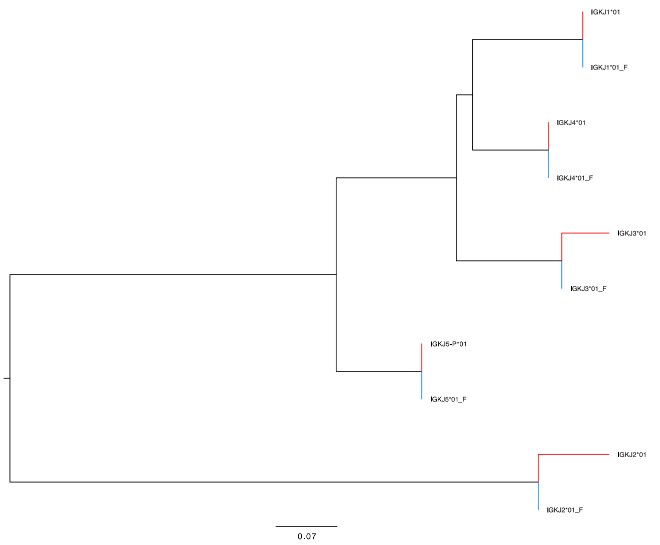

Fig. S5A, B: Maximum likelihood phylogenetic tree of human and macaque functional (A) IGLJ (B) IGLC genes. Human Ig genes are represented in blue and macaque Ig genes in red.

5A

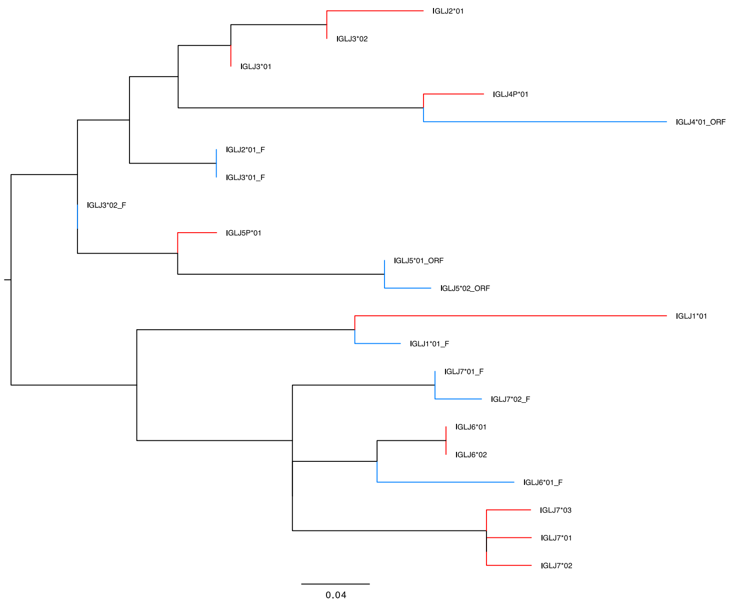

5B

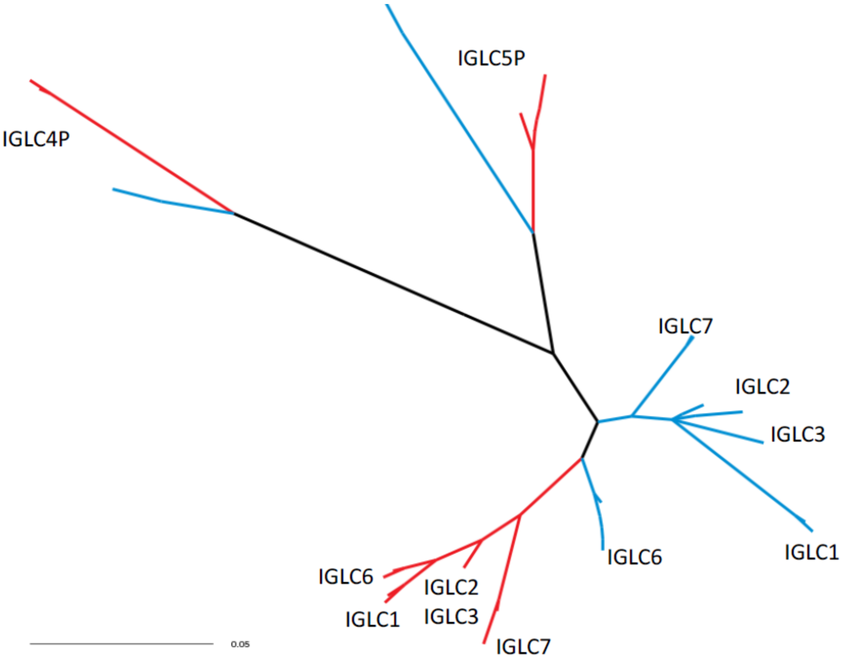

Fig. S6A, B: Maximum likelihood phylogenetic tree of human (6A) IGHV3 and (6B) IGLV3 genes. The position of the V gene on the human chromosome is represented after the hyphen.

6A

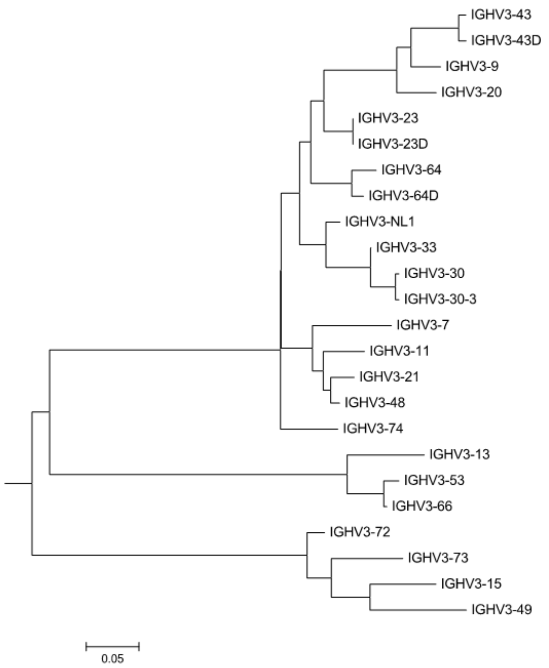

6B

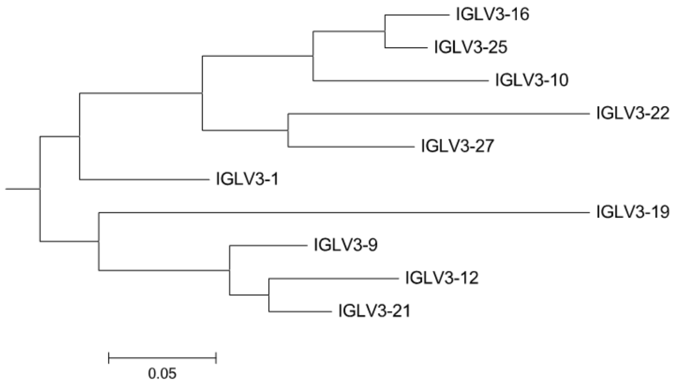

Fig S7: Signature of gene conversion

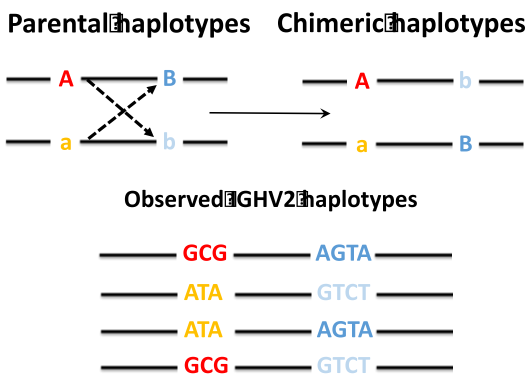

2.2 Supplementary Tables

Tables S1A, S1B: S1A: List of all IGHV genes (functional, non-functional, ORF) and (S1B) IGHD identified in the reference Ig macaque sequence

1A

IGHV family	number F/NF/ORF genes	number of F genes		
		location known	location unknown	sister alleles
IGHV1	28	3	1	2
IGHV2	8	2	-	3
IGHV3	84	28	1	7
IGHV4	35	7	5	6
IGHV5	4	2	-	-
IGHV6	2	1	-	-
IGHV7	17	1	-	2

1B

IGHD family	number of genes
IGHD1	5
IGHD2	7
IGHD3	7
IGHD4	7
IGHD5	7
IGHD6	6

Table S2: List of all IGKV genes (functional, non-functional, ORF) identified in the reference macaque Ig sequence

IGKV family	number F/NF/ORF genes	number of F genes	
		location known	sister alleles
IGKV1	39	20	5
IGKV2	47	24	1
IGKV3	14	6	5
IGKV4	4	1	-
IGKV6	3	2	-
IGKV7	1	1	-

Tables S3: List of all IGLV genes (functional, non-functional, ORF) identified in the reference macaque Ig sequence

IGLV family	number F/NF/ORF genes	number of F genes
**IGLV1**	15	10
**IGLV2**	18	9
**IGLV3**	29	8
**IGLV4**	4	1
**IGLV5**	19	8
**IGLV6**	5	3
**IGLV7**	8	4
**IGLV8**	2	1
**IGLV9**	2	-
**IGLV10**	2	1
**IGLV11**	1	1

Table S4: Assembly statistics for the nine rhesus macaques sequenced using baiting-Ig sequencing approach

Assembly statistics	Macaque 1	Macaque 2	Macaque 3	Macaque 4	Macaque 5	Macaque 6	Macaque 7	Macaque 8	Macaque 9
**# contigs >500bp**	8,584	8,478	8,271	8,678	8,563	8,448	8,599	11,232	8,565
**Largest contig (bp)**	34,790	35,005	19,262	25,878	32,473	19,036	33,183	24,227	33,193
**GC%**	44.21	44.06	43.7	43.89	43.91	43.87	43.53	42.87	43.77
**N50 (bp)**	2,411	2,423	1,751	2,089	2,409	2,313	2,011	1,403	2,124
**L50**	1,713	1,671	1,882	1,857	1,658	1,696	1,905	2,665	1,833

Tables S5A, S5B: S5A: List of all functional/ORF IGHV genes identified in the 9 rhesus macaques S5B: List of the total number of IGHC gene sequences and unique alleles (in parenthesis) found in the 9 rhesus macaques.

S5A

IGHV family	number F genes	number ORF genes
IGHV1	20	-
IGHV2	5	-
IGHV3	87	5
IGHV4	13	-
IGHV5	8	-
IGHV6	3	-
IGHV7	6	-

S5B

IGHC gene	CH1	CH2/H-CH2	CH3/CH3-CHS	CH4/CH4-CHS	H1	M1	M2
IGHA	5 (4)	4 (3)	3 (3)	-		6 (1)	-
IGHD	-	-	3 (1)	-	8 (1)	10 (1)	-
IGHE	7 (4)	7 (1)	6 (3)	11 (5)	-	3 (2)	-
IGHEP	-	2 (1)	5 (1)	2 (2)	-	-	-
IGHM	6 (4)	7 (3)	11 (2)	9 (5)	-	-	-
IGHG	10 (4)	11 (5)	13 (7)	-	5 (2)	13 (1)	9 (2)

Tables S6A,B: List of all functional/ORF (S6A) IGKV (S6B) IGLV genes identified in the 9 rhesus macaques

S6A

IGKV family	number F genes	number ORF genes
IGKV1	64	1
IGKV2	75	5
IGKV3	26	-
IGKV4	1	-
IGKV5	5	-
IGKV6	4	-
IGKV7	2	-

S6B

IGLV family	number F genes	number ORF genes
IGLV1	30	-
IGLV2	13	-
IGLV3	35	-
IGLV4	4	-
IGLV5	18	-
IGLV6	10	-
IGLV7	9	2
IGLV8	3	3
IGLV9	-	-
IGLV10	1	-
IGLV11	2	-

Tables S7A-E: List of human (IMGT) and macaque (S7A) IGHV (S7B) IGHC (S7D) IGKV (S7E) IGLV genes and alleles. Table S7C lists pairwise identity of human and macaque IGHC exons

S7A

IGHV family	human F genes (alleles)	macaque F genes (alleles)
IGHV1	11 (39)	9 (25)
IGHV2	4 (37)	5 (9)
IGHV3	24 (103)	39 (115)
IGHV4	10 (78)	20 (31)
IGHV5	2 (9)	2 (8)
IGHV6	1 (2)	1 (3)
IGHV7	1 (5)	3 (7)

S7B

IGHC gene	human alleles	macaque alleles
IGHA1	2	4
IGHA2	3	-
IGHD	2	1
IGHE	4	5
IGHEP	2	3
IGHG1	5	3
IGHG2	6	2
IGHG3	19	3
IGHG4	4	3
IGHGP	2	-
IGHM	4	5

S7C

IGHC gene	CH1	CH2/H-CH2	CH3/CH3-CHS	CH4/CH4-CHS
IGHA	89.5, 87.5	95.4, 94.9	81.6, 79.1	-
IGHD	81.7	91.6	92.2	-
IGHE	89.9	89	92.6	93.9
IGHM	93	91.9	94.9	96.6
IGHG1	96.2	92.4	95.3	-
IGHG2	94.2	89.3	95	-
IGHG3	94.5	93	93.4	-
IGHG4	93.8	94.5	91.9	-

S7D

IGKV family	human F genes (alleles)	macaque F genes (alleles)
IGKV1	20 (33)	27 (80)
IGKV2	10 (17)	26 (88)
IGKV3	7 (13)	11 (33)
IGKV4	1 (1)	1 (1)
IGKV5	1 (1)	2 (5)
IGKV6	2 (2)	2 (5)
IGKV7	-	1 (2)

S7E

IGLV family	human F genes (alleles)	macaque F genes (alleles)
IGLV1	5 (9)	10 (35)
IGLV2	5 (17)	9 (18)
IGLV3	10 (17)	12 (38)
IGLV4	3 (6)	1 (4)
IGLV5	4 (8)	8 (19)
IGLV6	1 (2)	3 (11)
IGLV7	2 (3)	4 (9)
IGLV8	1 (3)	1 (3)
IGLV9	1 (3)	-
IGLV10	1 (3)	1 (1)
IGLV11	-	1 (2)

Table S8: Signatures of gene conversion in macaque Ig V genes. Monkey 0 indicates that the Ig V gene was identified in the high quality Ig assembly. Monkey 1-9 indicates that the Ig V genes were identified from the Ig-bait sequencing on the nine macaques

Macaque ID	Ig V gene	position1/nt	position2/nt
Macaque 3	IGHV2-ABG*02	177-179, GCG	231-240-243-246, AGTA
Macaque 0	IGHV2-ABU*01	177-179, GCG	231-240-243-246, GTCT
Macaque 6	IGHV2-AEY*02	177-179, ATA	231-240-243-246, AGTA
Macaque 4,5,8	IGHV2-ACK*02	177-179, ATA	231-240-243-246, GTCT
Macaque 3	IGHV1-AGS*04	1-5-6, GTC	69-71-75-83-84-..-255-272, GTCCC-..-CC
Macaque 2	IGHV1-AGS*03	1-5-6, CAG	69-71-75-83-84-..-255-272, GTCCC-..-CC
Macaque 1,5,8	IGHV1-AHB*01	1-5-6, CAG	69-71-75-83-84-..-255-272, ACTTT-..-TT
Macaque 9	IGHV1-AAU*03	1-5-6, GTC	69-71-75-83-84-..-255-272, ACTTT-..-TT

Macaque 3,4,5,8	IGLV8-AEE*01	40-82-83, TCT	143-244, CA
Macaque 2,5	IGLV8-AEE*02	40-82-83, TCT	143-244, TG
Macaque 8,2	IGLV8-ABK*02	40-82-83, CTC	143-244, TG
Macaque 7	IGLV8-ABK*03	40-82-83, CTC	143-244, CA

Table S9: Celerav8.1 parameters used to assemble the SLRs generated from Illumina TruSeq strategy

unitigger=bogart
merSize=31
merThreshold=auto*2
ovlMinLen=300
obtErrorRate=0.03
obtErrorLimit=4.5
ovlErrorRate=0.03
utgErrorRate=0.015
utgGraphErrorRate=0.015
utgGraphErrorLimit=0
utgMergeErrorRate=0.03
utgMergeErrorLimit=0
